# Drug utilization pattern of romosozumab and other osteoporosis treatments in Japan, 2019–2021

**DOI:** 10.1007/s00774-024-01530-6

**Published:** 2024-07-10

**Authors:** Satoshi Soen, Alex Wang, Etsuro Hamaya, Hsu-Chih Chien, Tzu-Chieh Lin

**Affiliations:** 1 Soen Orthopedics, Osteoporosis, and Rheumatology Clinic, 2-14-10 Okamoto, Higashinada-Ku, Kobe, Hyogo 658-0072 Japan; 2 Medical Development, Amgen Inc, Sydney, Australia; 3 Medical Affairs, Amgen K.K, Tokyo, Japan; 4 Center for Observational Research, Amgen Inc, Thousand Oaks, CA USA

**Keywords:** Comorbidity, Japan, Osteoporosis, Osteoporotic fracture, Romosozumab

## Abstract

**Introduction:**

Describe real-world treatment of osteoporosis and romosozumab treatment patterns in Japan.

**Materials and methods:**

Data for patients initiating romosozumab or other antiosteoporotic medications between March 01, 2018, and May 31, 2022, were extracted from the Medical Data Vision (MDV) and Japan Medical Data Center (JMDC) databases. Patients were categorized into four cohorts: those who newly initiated romosozumab within the first (MDV: n = 4782; JMDC: n = 2578) or second (MDV: n = 3888; JMDC: n = 2446) year after launch and those who initiated teriparatide (TPTD; MDV: n = 14,576; JMDC: n = 8259) or non-TPTD antiosteoporotic medications within the first year of romosozumab launch (MDV: n = 352,142; JMDC: n = 185,785).

**Results:**

Mean age, sex, baseline cardiovascular history, comorbidities, and concomitant medications were similar across cohorts. In the MDV database, fracture history was higher in the romosozumab year-1 (59.3%), year-2 (64.1%), and TPTD (65.5%) cohorts versus the non-TPTD cohort (24.4%). Similar rates were identified in the JMDC database: romosozumab year-1 (64.7%), year-2 (66.6%), TPTD (67.5%), and non-TPTD (27.8%). Vertebral fractures were most common in all cohorts. 12-month romosozumab discontinuation varied between the year-1 and year-2 cohorts in MDV (62.4% and 58.8%) and JMDC (57.1% and 52.7%), whereas mean number of injections remained consistent (MDV: 9.7 and 9.8; JMDC: 7.3 and 7.8). Romosozumab persistence was lower in year-1 versus year-2 (MDV: 37.6% and 42.9%; JMDC: 41.2% and 47.3%).

**Conclusion:**

Patients initiating romosozumab and TPTD had a high fracture history. Given the dual effects of promoting bone formation and suppressing resorption, improving romosozumab adherence and persistence over time may be important for antiosteoporotic therapy.

**Supplementary Information:**

The online version contains supplementary material available at 10.1007/s00774-024-01530-6.

## Introduction

Osteoporosis is a skeletal disorder characterized by diminished bone strength, which predisposes affected individuals to an increased risk of fracture [1]. The risk of developing osteoporosis increases with age, with significant long-term adverse outcomes anticipated for elderly populations [1, 2]. In Japan, a country with one of the highest life expectancy rates in the world [3], the Japan National Health and Wellness survey (2012–2014) estimated that fractures affected 37.4% of individuals with osteoporosis aged ≥ 50 years, of whom 41.5% had multiple fractures [4]. Indeed, osteoporotic fractures have been associated with a 1.86-fold increase in the risk of subsequent fractures [5]. Vertebrae are the most common fracture site in the Japanese population, with an estimated prevalence of 25% among those in their early 70s, increasing to 43% among those aged > 80 years [6]. Hip fractures are also very common and had previously been estimated to affect approximately 5.11 in 10,000 men and 18.14 in 10,000 women in Japan in 2007 [7–10].

The management of osteoporosis includes the administration of antiresorptive agents with various mechanisms of action and osteoanabolic or bone-forming agents (i.e., teriparatide [TPTD] and romosozumab) [6, 11]. The efficacy and safety of romosozumab, a recombinant humanized antisclerostin monoclonal antibody that promotes bone formation and decreases bone resorption by neutralizing the suppression of Wnt signaling [12], have been demonstrated in studies of postmenopausal women with osteoporosis and men [13–17]. Romosozumab was approved in Japan in January 2019 for the treatment of osteoporosis in patients at high risk of fracture, as defined by the World Health Organization’s Fracture Risk Assessment Tool (FRAX®) and the Japanese Society for Bone and Mineral Research and Japan Osteoporosis Society [13, 18, 19]. Importantly, the romosozumab label was updated in April 2019 to include up-to-date information regarding the risks associated with cardiovascular (CV) disease, and the Japan Risk Management Plan continues to monitor the associated risks via pharmacovigilance periodic reports [20].

Despite multiple available therapeutic strategies, only 20% of patients with osteoporosis receive treatment [14], with 16.6% of those with hip fractures and 34.6% of those with vertebral fractures treated following discharge, despite the high risk of subsequent fractures [15]. In addition, persistence to osteoporosis therapy in Japan has been low, with persistence rates of only 46.3% and a medication possession ratio (MPR) of only 61.9% after 2 years, resulting in poor fracture control [16]. Measures to effectively manage osteoporosis are urgently needed in the rapidly aging population of Japan [11, 17].

Importantly, the Japanese Guideline for the Prevention and Treatment of Osteoporosis does not specify the preference for one treatment over another with the same indication [21]. As such, in a real-world setting, the choice of drug is likely indicative of other important considerations, such as underlying CV risk or the risk of osteoporotic fracture. The selected drug may therefore serve as a marker of a high risk of osteoporotic fracture or CV events, by proxy. Indeed, recent evidence suggests that patients with a high risk of fracture may particularly benefit from a treatment sequence comprising romosozumab followed by antiresorptive therapy [22] Additionally, romosozumab carries a black box warning for patients who have experienced myocardial infarction or stroke within the past year [22]. Further research is thus needed to optimize the treatment sequence and assess the safety of romosozumab, particularly in patients with CV risk factors.

This real-world, longitudinal, retrospective study of two Japanese administrative databases aimed to assess the clinical utility and baseline characteristics of patients with osteoporosis who initiated romosozumab versus TPTD or non-TPTD therapy within the first 2 years after romosozumab launch. Patients who newly initiated romosozumab within the first year after its launch in Japan were compared with those who newly initiated romosozumab within the second year after launch (after the label change) and those who initiated other antiosteoporotic medications (TPTD or non-TPTD) within the first year after romosozumab launch.

## Materials and methods

### Data source

Data from the Japan Medical Data Center (JMDC) hospital database and the Medical Data Vision (MDV) database were analyzed. Although both databases contain claims data, the characteristics of their associated populations differ; therefore, each database was analyzed separately. The JMDC is one of Japan’s largest claims databases and contains epidemiological data available for academic and industry use. It comprises claims receipts (inpatient, outpatient, and pharmacy) and Diagnosis Procedure Combination (DPC) medical data, accumulated from health insurance associations since 2005. Inpatient and outpatient visit data are available from 2014 onward [23]. Claims and administrative data are available from approximately 200 medical institutions across Japan, including 10% of direct and nondirect primary care hospitals, for approximately 17 million patients. As JMDC claims data are provided by insurance associations, patient transfers across hospitals may be tracked over time. However, as the JMDC incorporates health insurance association claims, it has a lower proportion of patients aged 65 or over [24]. The MDV database is an electronic health records–based database containing DPC data, anonymized health insurance claims information (social, national, and elderly insurance), and administrative data sourced from over 480 hospitals. The MDV database contains status and treatment data for approximately 45 million patients across Japan, of whom a significant proportion are over the age of 65 years [25]. Analyses of MDV data are benefited by the ability to assess diseases commonly affecting elderly populations [26].

### Study design

This retrospective, longitudinal study collected data from the MDV and JMDC databases between March 1, 2018, and May 31, 2022. Patients with osteoporosis who newly initiated romosozumab or other TPTD or non-TPTD antiosteoporotic medications in the first year after romosozumab launch in Japan (March 1, 2019, to February 29, 2020) and romosozumab in the second year after launch (March 1, 2020, and February 28, 2021) were identified. The index date was defined as the initiation date of romosozumab, other TPTD, or non-TPTD antiosteoporotic medications (Online Resource Fig. S1). The baseline period was defined as 1 year prior to the index date. Patients were followed up until death, disenrollment, 18 months of follow-up, or the final date of the study period (May 31, 2022), whichever occurred first. Disenrollment was defined as no treatment record for 6 months since the last visit, and the date of disenrollment was considered the date of the last visit. Treatment patterns were reported in patients with at least 12 months of follow-up data.

### Patients

Eligible patients were aged ≥ 18 years on the index date, had at least one osteoporosis diagnosis during the baseline period or at the index date, and required at least one hospital visit during the baseline period. Osteoporosis diagnoses were determined using the International Classification of Diseases, Tenth Revision (ICD-10) codes M80.xx and M81.xx. Eligible patients who started romosozumab were categorized into two mutually exclusive cohorts: (i) those who newly initiated romosozumab in the first year after launch (romosozumab year-1 cohort), and (ii) those who newly initiated romosozumab in the second year after launch (romosozumab year-2 cohort). Patients who started antiosteoporotic medications other than romosozumab between March 1, 2019, and February 29, 2020, were further categorized into two cohorts according to the first antiosteoporotic medication received: (iii) those who started a TPTD (recombinant daily [QD], acetate weekly [QW], or twice weekly [2QW]), and (iv) those who started a non-TPTD. TPTD users were considered a proxy for a population of patients at high risk of osteoporotic fracture.

### Objectives and purpose

The primary objective of this analysis was to describe the demographic characteristics, medical history, fracture history, and medication use of patients who initiated romosozumab within the first versus second year of romosozumab launch in Japan, as well as those who initiated TPTD or non-TPTD antiosteoporotic medications within the first year of romosozumab launch. The secondary objective was to describe the clinical utility and treatment patterns including exposure, discontinuation, persistence, and adherence of romosozumab use compared with TPTD or non-TPTD therapy options during the 2 years after romosozumab launch. A full dose of romosozumab requires the monthly administration of two subcutaneous injections of a 105 mg/1.17 mL solution in single-use prefilled syringes; therefore, a full dose (210 mg) was considered valid for treatment pattern evaluation.

### Endpoints

The endpoints that informed the primary objective included (i) demographic characteristics at the index date, (ii) osteoporotic fracture history during the baseline period and at the index date, (iii) CV disease during the baseline period and at the index date, (iv) comorbidities during the baseline period and at the index date, (v) other concomitant medication use at the index date ± 2 weeks, and (vi) other antiosteoporotic medications used during the baseline period and at the index date for both romosozumab cohorts and at the index date only for the TPTD and non-TPTD cohorts. A full description of CV disease, concomitant medications, comorbidities, and other antiosteoporotic medications collected during the baseline period and at the index date is provided in Online Resource Table S1. Endpoints informing the secondary endpoint included (i) the number of romosozumab doses per patient and covered days (30 days covered per dose), (ii) patient discontinuation of romosozumab (defined as failure to receive romosozumab within 60 days after the previous dose), (iii) patient persistence with romosozumab (defined as staying on romosozumab without any discontinuation during follow-up), and (iv) adherence to romosozumab therapy (measured by the proportion of days covered during follow-up). Patients who discontinued romosozumab and subsequently restarted ≥ 60 days after the previous injection were also recorded. Only the first discontinuation and restart episodes were captured in this study.

### Statistical analysis

Continuous variables are presented as means, medians, ranges, and standard deviations (SDs) and categorical variables as frequency distributions. Differences in means and proportions among patient demographics, clinical characteristics, fracture history, and treatment use are presented using 95% confidence intervals (CIs) for the following pairwise exposure status: (i) romosozumab year-1 versus year-2 cohort; (ii) romosozumab year-1 versus TPTD cohort; and (iii) romosozumab year-1 versus non-TPTD cohort. No statistical imputation was performed to address missing data. Data were analyzed using SAS version 9 (SAS institute, Cary, North Carolina, USA).

## Results

### Patient disposition

The final analysis cohorts included in the study were (i) the romosozumab year-1 cohort (MDV: n = 4782; JMDC: n = 2578), (ii) the romosozumab year-2 cohort (MDV: n = 3888; JMDC: n = 2446), (iii) the TPTD cohort (MDV: n = 14,576; JMDC: n = 8259), and (iv) the non-TPTD cohort (MDV: n = 352,142; JMDC: n = 185,785; Online Resource Fig. S2).

### Demographics and baseline clinical characteristics

Age was similar between the cohorts. In the MDV database, the mean ages (SD) were 76.7 (9.0) years in the year-1 cohort, 76.8 (9.3) years in the year-2 cohort, 76.7 (9.2) years in the TPTD cohort, and 72.9 (13.1) years in the non-TPTD cohort, and in the JMDC database, the mean ages (SD) were 78.0 (9.0), 77.9 (9.0), 77.7 (9.4), and 74.4 (12.7) years, respectively. All cohorts predominantly comprised female (> 80%). At the index date, patients were predominantly captured via outpatient visits in the MDV (year-1: 93.2%, year-2: 93.0%, TPTD: 77.5%, non-TPTD: 84.7%) and JMDC (year-1: 77.9%, year-2: 82.7%, TPTD: 63.1%, non-TPTD: 81.0%; Table 1) databases.

**Table 1:** Demographics and baseline clinical characteristics

	Romosozumab year-1	Romosozumab year-2	Mean difference (year-1 vs year-2) % (95% CI)	TPTD	Mean difference (year-1 vs TPTD) % (95% CI)	Non-TPTD	Mean difference (year-1 vs non-TPTD) % (95% CI)
MDV database							
Patients, N	4782	3888	–	14,576	–	352,142	–
Mean age, years (SD)	76.7 (9.0)	76.8 (9.3)	− 0.1 (− 0.8, 0.7)	76.7 (9.2)	− 0.0 (− 0.6,0.6)	72.9 (13.1)	3.8 (3.3, 4.3)
Age group, years, n (%)							
< 50	45 (0.9)	47 (1.2)	− 0.3 (− 0.7, 0.2)	139 (1.0)	0.0 (− 0.3, 0.3)	22,006 (6.2)	− 5.3 (− 5.6, − 5.0)
50–59	159 (3.3)	142 (3.7)	− 0.3 (− 1.1, 0.5)	491 (3.4)	− 0.0 (− 0.6, 0.6)	26,212 (7.4)	− 4.1 (− 4.6, − 3.6)
60–69	692 (14.5)	537 (13.8)	0.7 (− 0.8, 2.2)	2206 (15.1)	− 0.7 (− 1.8, 0.5)	66,010 (18.7)	− 4.3 (− 5.3, − 3.3)
70–79	1912 (40.0)	1512 (38.9)	1.1 (− 1.0, 3.2)	5776 (39.6)	0.4 (− 1.3, 2.0)	117,391 (33.3)	6.6 (5.2, 8.1)
80–89	1748 (36.6)	1462 (37.6)	− 1.0 (− 3.1, 1.0)	5168 (35.5)	1.1 (− 0.5, 2.7)	101,122 (28.7)	7.8 (6.5, 9.2)
≥ 90	226 (4.7)	188 (4.8)	− 0.1 (− 1.0, 0.8)	796 (5.5)	− 0.7 (− 1.5, − 0.0)	19,401 (5.5)	− 0.8 (− 1.4, − 0.2)
Sex, n (%)							
Female	4213 (88.1)	3411 (87.7)	0.4 (− 1.0, 1.8)	11,957 (82.0)	6.1 (4.9, 7.2)	284,154 (80.7)	7.4 (6.5, 8.3)
Male	569 (11.9)	477 (12.3)	− 0.4 (− 1.8, 1.0)	2619 (18.0)	− 6.1 (− 7.2, − 4.9)	67,988 (19.3)	− 7.4 (− 8.3, − 6.5)
Settings, n (%)							
Inpatient	326 (6.8)	272 (7.0)	− 0.2 (− 1.3, 0.9)	3280 (22.5)	− 15.7 (− 16.7, − 14.7)	53,898 (15.3)	− 8.5 (− 9.2, − 7.8)
Outpatient	4456 (93.2)	3616 (93.0)	0.2 (− 0.9, 1.3)	11,296 (77.5)	15.7 (14.7, 16.7)	298,244 (84.7)	8.5 (7.8, 9.2)
JDMC database							
Patients, N	2578	2446	–	8259	–	185,785	–
Mean age, years (SD)	78.0 (9.0)	77.9 (9.0)	0.0 (− 0.9, 1.0)	77.7 (9.4)	0.2 (− 0.5, 1.0)	74.4 (12.7)	3.6 (2.9, 4.3)
Age group, years, n (%)							
< 50	22 (0.9)	25 (1.0)	− 0.2 (− 0.7, 0.4)	75 (0.9)	− 0.1 (− 0.5, 0.4)	9330 (5.0)	− 4.2 (− 4.6, − 3.8)
50–59	69 (2.7)	67 (2.7)	− 0.1 (− 1.0, 0.9)	250 (3.0)	− 0.4 (− 1.1, 0.4)	11,535 (6.2)	− 3.5 (− 4.2, − 2.9)
60–69	307 (11.9)	271 (11.1)	0.8 (− 1.0, 2.6)	1110 (13.4)	− 1.5 (− 3.0, − 0.1)	32,210 (17.3)	− 5.4 (− 6.7, − 4.1)
70–79	953 (37.0)	923 (37.7)	− 0.8 (− 3.5, 1.9)	3019 (36.6)	0.4 (− 1.7, 2.6)	60,603 (32.6)	4.3 (2.5, 6.2)
80–89	1057 (41.0)	1000 (40.9)	0.1 (− 2.6, 2.9)	3116 (37.7)	3.3 (1.1, 5.5)	58,991 (31.8)	9.2 (7.3, 11.2)
≥ 90	170 (6.6)	160 (6.5)	0.1 (− 1.4, 1.5)	689 (8.3)	− 1.7 (− 2.9, − 0.6)	13,116 (7.1)	− 0.5 (− 1.5, 0.5)
Sex, n (%)							
Female	2258 (87.6)	2135 (87.3)	0.3 (− 1.6, 2.2)	6850 (82.9)	4.6 (3.1, 6.2)	153,153 (82.4)	5.2 (3.8,6.5)
Male	320 (12.4)	311 (12.7)	− 0.3 (− 2.2, 1.6)	1409 (17.1)	− 4.6 (− 6.2, − 3.1)	32,632 (17.6)	− 5.2 (− 6.5, − 3.8)
Settings, n (%)							
Inpatient	571 (22.1)	424 (17.3)	4.8 (2.6, 7.0)	3051 (36.9)	− 14.8 (− 16.7, − 12.9)	35,376 (19.0)	3.1 (1.5,4.7)
Outpatient	2007 (77.9)	2022 (82.7)	− 4.8 (− 7.0, − 2.6)	5208 (63.1)	14.8 (12.9, 16.7)	150,409 (81.0)	− 3.1 (− 4.7, − 1.5)

*CI* confidence interval, *JMDC* Japan Medical Data Center, *max* maximum, *MDV* Medical Data Vision, *SD* standard deviation, *TPTD* teriparatide

### Fracture history

High rates of osteoporotic fracture were observed among patients in the MDV database in the year-1 (59.3%), year-2 (64.1%), and TPTD (65.5%) cohorts, whereas a lower rate was observed in the non-TPTD (24.4%) cohort. The most common fracture site across all cohorts was the vertebra (72.3%, 70.3%, 70.9%, and 57.0%, respectively). In the JMDC database, similar rates were observed in the year-1 (64.7%), year-2 (66.6%), and TPTD (67.5%) cohorts, with a lower rate in the non-TPTD (27.8%) cohort. The most common fracture site was the vertebra (67.3%, 70.6%, 66.0%, and 58.6%, respectively). The proportion of patients with hip fractures was similar across all cohorts in both databases (Table 2).

**Table 2:** Fracture history during the baseline period and at the index date

	Romosozumab year-1	Romosozumab year-2	Mean difference (year-1 vs year-2) % (95% CI)	TPTD	Mean difference (year-1 vs TPTD) % (95% CI)	Non-TPTD	Mean difference (year-1 vs non-TPTD) % (95% CI)
MDV database							
Patients, N	4782	3888	–	14,576	–	352,142	–
Osteoporotic fracture, n (%)	2838 (59.3)	2493 (64.1)	− 4.8 (− 6.8, − 2.7)	9552 (65.5)	− 6.2 (− 7.8, − 4.6)	85,825 (24.4)	35.0 (33.6, 36.4)
Site of fracture^a^, n (%)							
Vertebral	2051 (72.3)	1753 (70.3)	2.0 (− 0.5, 4.4)	6772 (70.9)	1.4 (− 0.5, 3.3)	48,944 (57.0)	15.2 (13.5, 16.9)
Hip	537 (18.9)	474 (19.0)	− 0.1 (− 2.2, 2.1)	1667 (17.5)	1.5 (− 0.2, 3.1)	21,373 (24.9)	− 6.0 (− 7.5, − 4.5)
Other	743 (26.2)	687 (27.6)	− 1.4 (− 3.8, 1.0)	2465 (25.8)	0.4 (− 1.5, 2.2)	27,022 (31.5)	− 5.3 (− 7.0, − 3.6)
JMDC database							
Patients, N	2578	2446	–	8259	–	185,785	–
Osteoporotic fracture, n (%)	1668 (64.7)	1629 (66.6)	− 1.9 (− 4.6, 0.8)	5571 (67.5)	− 2.8 (− 4.9, − 0.6)	51,625 (27.8)	36.9 (35.0, 38.8)
Site of fracture^a^, n (%)							
Vertebral	1123 (67.3)	1150 (70.6)	− 3.3 (− 6.5, − 0.1)	3678 (66.0)	1.3 (− 1.3, 3.9)	30,245 (58.6)	8.7 (6.4, 11.1)
Hip	323 (19.4)	305 (18.7)	0.6 (− 2.1, 3.4)	1136 (20.4)	− 1.0 (− 3.2, 1.2)	12,536 (24.3)	− 4.9 (− 6.9, − 3.0)
Other	467 (28.0)	437 (26.8)	1.2 (− 1.9, 4.3)	1505 (27.0)	1.0 (− 1.5, 3.5)	15,627 (30.3)	− 2.3 (− 4.5, − 0.1)

*CI* confidence interval, *JMDC* Japan Medical Data Center, *MDV* Medical Data Vision, *TPTD* teriparatide

^a^Percentages calculated using the number of patients with osteoporotic fractures as the denominator. The percentages sum to over 100% as some patients could have experienced fractures at multiple sites prior to the index date

### CV disease history

A full description of CV disease history during the baseline period is provided in Table 3 and Online Resource Table S2. The most common CV diseases in the MDV database were angina pectoris (year-1: 11.5%, year-2: 7.9%, TPTD: 11.5%, and non-TPTD: 12.7%) and cerebral infarction (year-1: 6.1%, year-2: 4.7%, TPTD: 5.8%, and non-TPTD: 6.5%). Similarly, in the JMDC database, angina pectoris was the most common (year-1: 11.8%, year-2: 10.0%, TPTD: 11.6%, and non-TPTD: 13.9%), followed by cerebral infarction (year-1: 6.1%, year-2: 4.6%, TPTD: 5.5%, and non-TPTD: 7.1%). Sequelae of cerebrovascular disease were frequent in both the MDV (year-1: 5.2%, year-2: 4.3%, TPTD: 5.2%, and non-TPTD: 4.9%) and JMDC (year-1: 5.2%, year-2: 4.9%, TPTD: 6.4%, and non-TPTD: 5.6%, respectively) databases.

**Table 3:** CV disease history during the baseline period and at the index date

CV disease, n (%)	Romosozumab year-1	Romosozumab year-2	Mean difference (year-1 vs year-2) % (95% CI)	TPTD	Mean difference (year-1 vs TPTD) % (95% CI)	Non-TPTD	Mean difference (year-1 vs non-TPTD) % (95% CI)
MDV database							
Patients, N	4782	3888	–	14,576	–	352,142	–
Ischemic heart disease	610 (12.8)	347 (8.9)	3.8 (2.5, 5.2)	1871 (12.8)	0.0 (− 1.1, 1.0)	49,990 (14.2)	− 1.4 (− 2.4, − 0.5)
Cerebrovascular disease	673 (14.1)	432 (11.1)	3.0 (1.5, 4.4)	1856 (12.7)	1.3 (0.2, 2.5)	49,019 (13.9)	0.2 (− 0.8, 1.2)
Procedure	12 (0.3)	5 (0.1)	0.1 (− 0.1,0.3)	48 (0.3)	− 0.1 (− 0.3, 0.1)	1663 (0.5)	− 0.2 (− 0.4, − 0.1)
PCI	11 (0.2)	5 (0.1)	0.1 (− 0.1, 0.3)	46 (0.3)	− 0.1 (− 0.3, 0.1)	1581 (0.4)	− 0.2 (− 0.4, − 0.1)
CABG	1 (< 0.1)	NA	NA	2 (< 0.1)	0.0 (− 0.1, 0.1)	91 (0.0)	0.0 (− 0.1, 0.0)
ACS event	4 (0.1)	2 (0.1)	0.0 (− 0.1, 0.2)	26 (0.2)	− 0.1 (− 0.2, 0.0)	1069 (0.3)	− 0.2 (− 0.3, − 0.1)
Stroke event	19 (0.4)	16 (0.4)	0.0 (− 0.3, 0.3)	92 (0.6)	− 0.2 (− 0.5, 0.0)	3240 (0.9)	− 0.5 (− 0.7, − 0.3)
JMDC database							
Patients, N	2578	2446	–	8259	–	185,785	–
Ischemic heart disease	327 (12.7)	277 (11.3)	1.4 (− 0.5, 3.2)	1082 (13.1)	− 0.4 (− 1.9, 1.1)	28,770 (15.5)	− 2.8 (− 4.1, − 1.5)
Cerebrovascular disease	345 (13.4)	308 (12.6)	0.8 (− 1.1, 2.7)	1148 (13.9)	− 0.5 (− 2.1, 1.0)	29,018 (15.6)	− 2.2 (− 3.6, − 0.9)
Procedure	5 (0.2)	2 (0.1)	0.1 (− 0.1, 0.4)	16 (0.2)	0.0 (− 0.2, 0.2)	583 (0.3)	− 0.1 (− 0.3, 0.1)
PCI	4 (0.2)	2 (0.1)	0.1 (− 0.2, 0.3)	15 (0.2)	0.0 (− 0.2, 0.2)	558 (0.3)	− 0.1 (− 0.3, 0.0)
CABG	1 (< 0.1)	NA	NA	1 (< 0.1)	0.0 (− 0.1, 0.1)	28 (< 0.1)	0.0 (− 0.1, 0.1)
ACS event	3 (0.1)	3 (0.1)	0.0 (− 0.2, 0.2)	7 (0.1)	0.0 (− 0.1, 0.2)	301 (0.2)	0.0 (− 0.2, 0.1)
Stroke event	15 (0.6)	20 (0.8)	− 0.2 (− 0.7, 0.3)	88 (1.1)	− 0.5 (− 0.9, − 0.1)	2346 (1.3)	− 0.7 (− 1.0, − 0.4)

*ACS* acute coronary syndrome, *CABG* coronary artery bypass graft, *CI* confidence interval, *CV* cardiovascular; *JMDC* Japan Medical Data Center, *MDV* Medical Data Vision, *MI* myocardial infarction, *NA* not available, *PCI* percutaneous coronary intervention, *TPTD* teriparatide

### Comorbidities

The most frequently recorded comorbidities for all cohorts in the MDV database were hypertension (year-1: 42.5%, year-2: 39.9%, TPTD: 43.2%, and non-TPTD: 45.5%), hyperlipidemia (year-1: 25.9%, year-2: 24.6%, TPTD: 25.9%, and non-TPTD: 33.9%), diabetes (year-1: 22.9%, year-2: 19.8%, TPTD: 23.4%, and non-TPTD: 30.8%), and peptic ulcer (year-1: 21.6%, year-2: 17.3%, TPTD: 18.1%, and non-TPTD: 24.0%). Similarly, in the JMDC database, common comorbidities included hypertension (year-1: 44.3%, year-2: 43.1%, TPTD: 46.7%, and non-TPTD: 48.1%), hyperlipidemia (year-1: 26.7%, year-2: 26.6%, TPTD: 26.5%, and non-TPTD: 35.0%), diabetes (year-1: 20.5%, year-2: 20.8%, TPTD: 22.5%, and non-TPTD: 29.6%), and peptic ulcer (year-1: 17.6%, year-2: 16.9%, TPTD: 16.3%, and non-TPTD: 22.6%). Other comorbidities included malignancy, chronic pulmonary disease, congestive heart failure, liver disease, peripheral vascular disease, chronic obstructive pulmonary disease, cardiac arrhythmia, and rheumatoid arthritis (Table 4).

**Table 4:** Comorbidities during the baseline period

	Romosozumab year-1	Romosozumab year-2	Mean difference (year-1 vs year-2) % (95% CI)	TPTD	Mean difference (year-1 vs TPTD) % (95% CI)	Non-TPTD	Mean difference (year-1 vs non-TPTD) % (95% CI)
MDV database							
Patients, N	4782	3888	–	14,576	–	352,142	–
HIV/AIDS	NA	1 (< 0.1)	NA	12 (0.1)	NA	421 (0.1)	NA
Any malignancy	531 (11.1)	465 (12.0)	− 0.9 (− 2.2, 0.5)	1178 (8.1)	3.0 (2.0, 4.0)	66,423 (18.9)	− 7.8 (− 8.7, − 6.8)
Diabetes	1094 (22.9)	769 (19.8)	3.1 (1.3, 4.8)	3404 (23.4)	− 0.5 (− 1.9, 0.9)	108,549 (30.8)	− 7.9 (− 9.2, − 6.7)
Diabetes complications	229 (4.8)	137 (3.5)	1.3 (0.4, 2.1)	667 (4.6)	0.2 (− 0.5, 0.9)	19,679 (5.6)	− 0.8 (− 1.4, − 0.2)
Hyperlipidemia	1238 (25.9)	958 (24.6)	1.2 (− 0.6, 3.1)	3773 (25.9)	0.0 (− 1.4, 1.4)	119,358 (33.9)	− 8.0 (− 9.3, − 6.7)
Dementia	305 (6.4)	270 (6.9)	− 0.6 (− 1.6, 0.5)	870 (6.0)	0.4 (− 0.4, 1.2)	18,518 (5.3)	1.1 (0.4, 1.8)
Parkinson’s disease	126 (2.6)	90 (2.3)	0.3 (− 0.4, 1.0)	430 (3.0)	− 0.3 (− 0.9, 0.2)	6849 (1.9)	0.7 (0.2, 1.2)
Hemiplegia/paraplegia	34 (0.7)	26 (0.7)	0.0 (− 0.3, 0.4)	219 (1.5)	− 0.8 (− 1.1, − 0.5)	3211 (0.9)	− 0.2 (− 0.5, 0.0)
Hypertension	2030 (42.5)	1550 (39.9)	2.6 (0.5, 4.7)	6299 (43.2)	− 0.8 (− 2.4, 0.9)	160,292 (45.5)	− 3.1 (− 4.5, − 1.6)
Cardiac arrhythmias	474 (9.9)	321 (8.3)	1.7 (0.4, 2.9)	1671 (11.5)	− 1.6 (− 2.6, − 0.5)	38,479 (10.9)	− 1.0 (− 1.9, − 0.2)
CHF	670 (14.0)	446 (11.5)	2.5 (1.1, 4.0)	2037 (14.0)	0.0 (− 1.1, 1.2)	59,405 (16.9)	− 2.9 (− 3.9, − 1.9)
Peripheral vascular disease	461 (9.6)	281 (7.2)	2.4 (1.2, 3.6)	1107 (7.6)	2.0 (1.1, 3.0)	33,075 (9.4)	0.2 (− 0.6, 1.1)
Chronic pulmonary disease	665 (13.9)	483 (12.4)	1.5 (0.0, 2.9)	1727 (11.8)	2.1 (0.9, 3.2)	62,863 (17.9)	− 3.9 (− 4.9, − 2.9)
COPD	335 (7.0)	280 (7.2)	− 0.2 (− 1.3, 0.9)	896 (6.1)	0.9 (0.0, 1.7)	34,169 (9.7)	− 2.7 (− 3.4, − 2.0)
Asthma	337 (7.0)	236 (6.1)	1.0 (− 0.1, 2.0)	906 (6.2)	0.8 (0.0, 1.7)	31,621 (9.0)	− 1.9 (− 2.7, − 1.2)
Peptic ulcer	1,034 (21.6)	674 (17.3)	4.3 (2.6, 6.0)	2642 (18.1)	3.5 (2.2, 4.8)	84,480 (24.0)	− 2.4 (− 3.6, − 1.2)
Liver disease	555 (11.6)	425 (10.9)	0.7 (− 0.7, 2.0)	1449 (9.9)	1.7 (0.6, 2.7)	53,054 (15.1)	− 3.5 (− 4.4, − 2.5)
Moderate/severe liver disease	19 (0.4)	24 (0.6)	− 0.2 (− 0.5, 0.1)	98 (0.7)	− 0.3 (− 0.5, 0.0)	2976 (0.8)	− 0.4 (− 0.6, − 0.3)
Rheumatoid arthritis	762 (15.9)	465 (12.0)	4.0 (2.5, 5.5)	1414 (9.7)	6.2 (5.1, 7.4)	71,196 (20.2)	− 4.3 (− 5.3, − 3.2)
SLE	84 (1.8)	50 (1.3)	0.5 (− 0.1, 1.0)	191 (1.3)	0.4 (0.0, 0.9)	13,437 (3.8)	− 2.1 (− 2.4, − 1.7)
Renal disease	434 (9.1)	245 (6.3)	2.8 (1.6, 3.9)	712 (4.9)	4.2 (3.3, 5.1)	31,082 (8.8)	0.2 (− 0.6, 1.1)
CKD	389 (8.1)	214 (5.5)	2.6 (1.6, 3.7)	604 (4.1)	4.0 (3.1, 4.8)	26,603 (7.6)	0.6 (− 0.2, 1.4)
CKD on dialysis	161 (3.4)	54 (1.4)	2.0 (1.3, 2.6)	92 (0.6)	2.7 (2.2, 3.3)	6498 (1.8)	1.5 (1.0, 2.0)
JMDC database							
Patients, N	2578	2446	–	8259	–	185,785	–
HIV/AIDS	NA	1 (< 0.1)	NA	NA	NA	189 (0.1)	NA
Any malignancy	233 (9.0)	238 (9.7)	− 0.7 (− 2.3, 1.0)	604 (7.3)	1.7 (0.5, 3.0)	31,355 (16.9)	− 7.8 (− 9.0, − 6.7)
Diabetes	528 (20.5)	508 (20.8)	− 0.3 (− 2.6, 2.0)	1861 (22.5)	− 2.1 (− 3.9, − 0.2)	55,039 (29.6)	− 9.1 (− 10.7, − 7.6)
Diabetes complications	95 (3.7)	80 (3.3)	0.4 (− 0.6, 1.5)	306 (3.7)	0.0 (− 0.9, 0.8)	9856 (5.3)	− 1.6 (− 2.4, − 0.9)
Hyperlipidemia	689 (26.7)	651 (26.6)	0.1 (− 2.4, 2.6)	2190 (26.5)	0.2 (− 1.8, 2.2)	65,048 (35.0)	− 8.3 (− 10.0, − 6.5)
Dementia	225 (8.7)	197 (8.1)	0.7 (− 0.9, 2.2)	730 (8.8)	− 0.1 (− 1.4, 1.2)	13,036 (7.0)	1.7 (0.6, 2.8)
Parkinson’s disease	77 (3.0)	57 (2.3)	0.7 (− 0.3, 1.6)	263 (3.2)	− 0.2 (− 1.0, 0.6)	3973 (2.1)	0.8 (0.2, 1.5)
Hemiplegia/paraplegia	27 (1.0)	17 (0.7)	0.4 (− 0.2, 0.9)	98 (1.2)	− 0.1 (− 0.6, 0.3)	1933 (1.0)	0.0 (− 0.4, 0.4)
Hypertension	1142 (44.3)	1055 (43.1)	1.2 (− 1.6, 4.0)	3860 (46.7)	− 2.4 (− 4.7, − 0.2)	89,278 (48.1)	− 3.8 (− 5.7, − 1.8)
Cardiac arrhythmias	240 (9.3)	223 (9.1)	0.2 (− 1.4, 1.8)	928 (11.2)	− 1.9 (− 3.3, − 0.6)	20,902 (11.3)	− 1.9 (− 3.1, − 0.8)
CHF	406 (15.7)	336 (13.7)	2.0 (0.0, 4.0)	1280 (15.5)	0.3 (− 1.4, 1.9)	33,392 (18.0)	− 2.2 (− 3.7, − 0.8)
Peripheral vascular disease	185 (7.2)	178 (7.3)	− 0.1 (− 1.6, 1.4)	554 (6.7)	0.5 (− 0.7, 1.6)	17,973 (9.7)	− 2.5 (− 3.5, − 1.5)
Chronic pulmonary disease	360 (14.0)	285 (11.7)	2.3 (0.4, 4.2)	1024 (12.4)	1.6 (0.0, 3.1)	33,593 (18.1)	− 4.1 (− 5.5, − 2.7)
COPD	179 (6.9)	168 (6.9)	0.1 (− 1.4, 1.5)	552 (6.7)	0.3 (− 0.9, 1.4)	17,499 (9.4)	− 2.5 (− 3.5, − 1.5)
Asthma	210 (8.1)	155 (6.3)	1.8 (0.3, 3.3)	533 (6.5)	1.7 (0.5, 2.9)	17,344 (9.3)	− 1.2 (− 2.3, − 0.1)
Peptic ulcer	455 (17.6)	413 (16.9)	0.8 (− 1.4, 2.9)	1345 (16.3)	1.4 (− 0.3, 3.1)	42,045 (22.6)	− 5.0 (− 6.5, − 3.5)
Liver disease	267 (10.4)	242 (9.9)	0.5 (− 1.2, 2.2)	843 (10.2)	0.1 (− 1.2, 1.5)	28,042 (15.1)	− 4.7 (− 5.9, − 3.5)
Moderate/severe liver disease	10 (0.4)	14 (0.6)	− 0.2 (− 0.6, 0.2)	62 (0.8)	− 0.4 (− 0.7, 0.0)	1381 (0.7)	− 0.4 (− 0.6, − 0.1)
Rheumatoid arthritis	257 (10.0)	187 (7.6)	2.3 (0.7, 3.9)	758 (9.2)	0.8 (− 0.5, 2.1)	31,896 (17.2)	− 7.2 (− 8.4, − 6.0)
SLE	20 (0.8)	21 (0.9)	− 0.1 (− 0.6, 0.5)	90 (1.1)	− 0.3 (− 0.7, 0.1)	6035 (3.2)	− 2.5 (− 2.8, − 2.1)
Renal disease	159 (6.2)	121 (4.9)	1.2 (− 0.1, 2.5)	377 (4.6)	1.6 (0.5, 2.7)	16,893 (9.1)	− 2.9 (− 3.9, − 2.0)
CKD	142 (5.5)	107 (4.4)	1.1 (− 0.1, 2.4)	321 (3.9)	1.6 (0.6, 2.6)	14,823 (8.0)	− 2.5 (− 3.4, − 1.6)
CKD on dialysis	35 (1.4)	13 (0.5)	0.8 (0.3, 1.4)	21 (0.3)	1.1 (0.6, 1.6)	2823 (1.5)	− 0.2 (− 0.6, 0.3)

*AIDS* acquired immunodeficiency syndrome, *CHF* congestive heart failure, *CI* confidence interval, *CKD* chronic kidney disease, *COPD* chronic obstructive pulmonary disease, *HIV* human immunodeficiency virus, *JMDC* Japan Medical Data Center, *MDV* Medical Data Vision, *NA* not available, *SLE* systemic lupus erythematosus, *TPTD* teriparatide

### Other concomitant medication use

Common concomitant medications used for all cohorts in both databases included angiotensin II receptor blockers, anticoagulants, calcium channel blockers, corticosteroids, cyclooxygenase-2 inhibitors, nonsteroidal antiinflammatory drugs, and statins (Online Resource Table S3). In the MDV database, similar proportions of the year-1, year-2, and TPTD cohorts received cyclooxygenase-2 inhibitors (11.8%, 14.5%, and 14.3%), corticosteroids (13.8%, 13.2%, and 13.1%), calcium channel blockers (12.5%, 12.1%, and 14.9%), and statins (9.4%, 8.1%, and 11.0%), respectively. A lower proportion of the non-TPTD cohort received cyclooxygenase-2 inhibitors (7.4%), whereas a higher proportion received corticosteroids (32.2%), calcium channel blockers (18.1%), and statins (17.8%). In the JMDC database, similar proportions of the year-1, year-2, and TPTD cohorts received cyclooxygenase-2 inhibitors (14.8%, 14.7%, and 17.0%), corticosteroids (11.4%, 11.5%, and 13.0%), calcium channel blockers (15.0%, 13.2%, and 17.9%), and statins (10.0%, 9.4%, and 11.1%), respectively. The use of cyclooxygenase-2 inhibitors was lower in the non-TPTD cohort (8.0%), whereas that of corticosteroids, calcium channel blockers, and statins was higher (28.0%, 19.6%, and 18.3%, respectively).

### Antiosteoporotic medication use in the romosozumab cohorts

In the year-1 and year-2 romosozumab cohorts, the most common antiosteoporotic medications used during the baseline period in the MDV database were active vitamin D_3_ (60.6% and 67.1%), which included alfacalcidol, calcitriol, and eldecalcitol, followed by bisphosphonates (43.1% and 42.3%), TPTD (22.5% and 18.5%), the receptor activator of nuclear factor kappa-Β ligand (RANKL) inhibitor denosumab (14.2% and 11.5%), and selective estrogen receptor modulators (SERMs; 7.5% and 8.8%), respectively. In the JMDC database, common antiosteoporotic medications included active vitamin D_3_ (65.0% and 66.1%), bisphosphonate (32.2% and 31.5%), TPTD (22.2% and 21.2%), the RANKL inhibitor denosumab (8.7% and 8.0%), and SERMs (7.6% and 7.5%), respectively. Among patients who had received TPTD, recombinant QD was the most common (MDV: 14.0% and 10.3%; JMDC: 11.8% and 13.1%, respectively), followed by acetate QW (MDV: 8.8% and 6.1%; JMDC: 10.6% and 6.1%, respectively; Table 5). Antiosteoporotic medication patterns based on days from initiation to the index date and days of supply are displayed in Online Resource Table S4.

**Table 5:** Antiosteoporotic medication use in the romosozumab cohort during the baseline period

Medications^a^	Romosozumab year-1		Romosozumab year-2		Mean difference (year-1 vs year-2) % (95% CI)	
	MDV	JMDC	MDV	JMDC	MDV	JMDC
Patients, N	4782	2578	3888	2446	–	–
Osteoporotic medication use, n (%)	3064 (64.1)	1430 (55.5)	2357 (60.6)	1220 (49.9)	–	–
Number of patients, n (%)^b^						
Bisphosphonate	1320 (43.1)	460 (32.2)	996 (42.3)	384 (31.5)	0.0 (− 0.1, 0.1)	0.0 (− 0.2, 0.2)
Calcitonin	114 (3.7)	87 (6.1)	70 (3.0)	69 (5.7)	0.0 (− 1.2,1.2)	0.0 (− 1.3, 1.3)
Calcium	190 (6.2)	84 (5.9)	121 (5.1)	39 (3.2)	0.0 (− 0.7, 0.7)	0.0 (− 1.9, 1.9)
Denosumab	436 (14.2)	125 (8.7)	270 (11.5)	97 (8.0)	0.0 (− 0.3, 0.3)	0.0 (− 0.9, 0.9)
Estrogen	4 (0.1)	1 (0.1)	6 (0.3)	2 (0.2)	0.0 (− 20.8, 20.8)	0.0 (− 75.0, 75.0)
Ipriflavone	4 (0.1)	–	NA	–	NA	–
SERM	229 (7.5)	108 (7.6)	207 (8.8)	92 (7.5)	0.0 (− 0.5, 0.5)	0.0 (− 1.0, 1.0)
TPTD (any of the below)	688 (22.5)	318 (22.2)	435 (18.5)	259 (21.2)	0.0 (− 0.2, 0.2)	0.0 (− 0.4, 0.4)
Recombinant QD	430 (14.0)	169 (11.8)	243 (10.3)	160 (13.1)	6.6 (0.5, 12.7)	− 8.6 (− 17.1, − 0.2)
Acetate QW	270 (8.8)	151 (10.6)	143 (6.1)	74 (6.1)	6.4 (0.5, 12.3)	18.9 (10.8, 27.0)
Acetate 2QW	1 (< 0.1)	3 (0.2)	55 (2.3)	32 (2.6)	− 12.5 (− 15.8, − 9.2)	− 11.4 (− 15.9, − 6.9)
Active vitamin D_3_^c^	1857 (60.6)	929 (65.0)	1581 (67.1)	806 (66.1)	0.0 (− 0.1, 0.1)	0.0 (− 0.1, 0.1)
Vitamin K	108 (3.5)	56 (3.9)	122 (5.2)	24 (2.0)	0.0 (− 0.9, 0.9)	0.0 (− 3.0, 3.0)
Methenolone acetate	NA	2 (0.1)	4 (0.2)	NA	NA	NA

*2QW* twice weekly; *CI* confidence interval; *JMDC* Japan Medical Data Center; *MDV* Medical Data Vision; *NA* not available; *QD* daily; *QW* weekly; *SERM* selective estrogen receptor modulator; *TPTD* teriparatide

^a^The medication may be used as monotherapy or in combination with other antiosteoporotic medications

^b^Percentages calculated using the total number of patients who received osteoporotic medication as the denominator

^c^Vitamin D3 included alfacalcidol, calcitriol, and eldecalcitol

### Romosozumab treatment patterns

Treatment patterns were reported only in romosozumab users with at least 12 months of follow-up time after the first dose. At 12 months of follow-up, the mean (SD) number of romosozumab doses received in the year-1 and year-2 cohorts was similar in the MDV (9.7 [5.1] and 9.8 [5.0]) and JMDC (7.3 [4.5] and 7.8 [4.3]) databases, as was the mean number of days covered in the MDV (258.0 and 261.0) and JMDC (210.3 and 224.5) databases. Discontinuation in the year-1 cohort was slightly higher than that in the year-2 cohort (MDV: 62.4% and 57.1%; JMDC: 58.8% and 52.7%) over the 12-month follow-up period. The mean adherence over 12 months for the year-1 cohort was similar to that for the year-2 cohort (MDV: 71.0% and 71.0%; JMDC: 58.0% and 61.0%). The treatment persistence rate at the 12-month follow-up was low for both cohorts (MDV: 37.6% and 42.9%; JMDC: 41.2% and 47.3%; Table 6).

**Table 6:** Romosozumab treatment patterns 12 months after the first dose

	Romosozumab year-1		Romosozumab year-2	
	MDV	JMDC	MDV	JMDC
Patients, N	4234	2155	3519	2106
Total romosozumab injections received				
Mean (SD)	9.7 (5.1)	7.3 (4.5)	9.8 (5.0)	7.8 (4.3)
Median (Q1, Q3)	12.0 (6.0, 12.0)	9.0 (2.0, 12.0)	12.0 (6.0, 12.0)	10.0 (3.0, 12.0)
Range (minimum, maximum)	1.0, 25.0	1.0, 14.0	1.0, 26.0	1.0, 14.0
Total days covered with romosozumab				
Mean (SD)	258.0 (117.4)	210.3 (129.0)	261.0 (117.2)	224.5 (124.1)
Median (Q1, Q3)	331.0 (150.0, 345.0)	256.0 (60.0, 342.0)	338.0 (171.0, 345.0)	286.0 (88.0, 342.0)
Range (minimum, maximum)	1.0, 389.0	10.0, 394.0	4.0, 389.0	14.0, 394.0
Patients who discontinued romosozumab^a^, n (%)	2643 (62.4)	1268 (58.8)	2008 (57.1)	1109 (52.7)
Patients who restarted romosozumab^b^, n (%)	452 (10.7)	341 (15.8)	296 (8.4)	271 (12.9)
Days from discontinuation to restart				
Mean (SD)	58.3 (35.5)	67.4 (49.4)	57.2 (36.0)	65.3 (52.2)
Median (Q1, Q3)	47.0 (33.0, 68.0)	54.0 (34.0, 68.0)	43.0 (33.0, 62.0)	46.0 (33.0, 68.0)
Range (minimum, maximum)	31.0, 266.0	31.0, 306.0	31.0, 234.0	31.0, 308.0
Patients persistent to romosozumab^c^ (crude), % (95% CI)	37.6 (35.9, 39.2)	41.2 (38.0, 44.3)	42.9 (41.0, 44.9)	47.3 (44.2, 50.5)
Patients adherent to romosozumab (proportion of days covered)^d^				
Mean MPR % (SD)	71.0 (32.0)	58.0 (35.0)	71.0 (32.0)	61.0 (34.0)
Median (Q1, Q3)	0.9 (0.4, 1.0)	0.7 (0.2, 1.0)	0.9 (0.5, 1.0)	0.8 (0.2, 0.9)
Range (minimum, maximum)	1.0 (0.0, 1.0)	1.0 (0.0, 1.0)	1.0 (0.0, 1.0)	1.0 (0.0, 1.0)
Percentage of days covered, n (%)				
Upper, lower 95% CI	0.7, 0.7	0.6, 0.6	0.7, 0.7	0.6, 0.6
0%–10%	373.0 (8.8)	345.0 (16.0)	317.0 (9.0)	281.0 (13.3)
10%–20%	242.0 (5.7)	233.0 (10.8)	195.0 (5.5)	179.0 (8.5)
20%–30%	203.0 (4.8)	135.0 (6.3)	136.0 (3.9)	115.0 (5.5)
30%–40%	192.0 (4.5)	113.0 (5.2)	166.0 (4.7)	110.0 (5.2)
40%–50%	173.0 (4.1)	116.0 (5.4)	144.0 (4.1)	119.0 (5.7)
50%–60%	94.0 (2.2)	65.0 (3.0)	81.0 (2.3)	65.0 (3.1)
60%–70%	99.0 (2.3)	63.0 (2.9)	75.0 (2.1)	81.0 (3.9)
70%–80%	233.0 (5.5)	149.0 (6.9)	163.0 (4.6)	151.0 (7.2)
80%–90%	461.0 (10.9)	215.0 (10.0)	326.0 (9.3)	282.0 (13.4)
90%–100%	2164.0 (51.1)	721.0 (33.5)	1916.0 (54.5)	723.0 (34.3)

*CI* confidence interval, *JMDC* Japan Medical Data Center, *MDV* Medical Data Vision, *MPR* medication possession ratio, *Q* quartile, *SD* standard deviation

^a^Discontinuation of romosozumab was defined as failure to receive the next dose within 60 days after the prior romosozumab dose

^b^Patients who discontinued and subsequently restarted romosozumab. Percentage of patients who restarted treatment was calculated according to the number of patients who discontinued treatment

^c^Patients who remained on romosozumab without any discontinuation from the time of the first romosozumab injection to the end of the covered days of the last observed romosozumab injection during follow-up

^d^Adherence to romosozumab was measured by the proportion of days covered during follow-up

## Discussion

Osteoporosis is a systemic disease that imposes a significant burden on the aging population worldwide in terms of morbidity, mortality, and healthcare costs associated with fractures [2]. The impact of fracture risk is amplified in Japan, a country with a fast-aging society and one of the highest life expectancies [3]. Romosozumab, an osteoanabolic drug that promotes bone formation and prevents resorption, is approved for increasing bone mineral density (BMD) and reducing the risk of fracture among men and postmenopausal women in Japan. This real-world, longitudinal, retrospective analysis evaluated Japanese patients with osteoporosis who received romosozumab, TPTD, or non-TPTD antiosteoporotic medications during the first or second year after the 2019 romosozumab launch in Japan. Patient characteristics and the clinical utility of antiosteoporotic drugs were investigated to determine the baseline clinical characteristics underlying real-world patterns of romosozumab prescription compared with TPTD and non-TPTD options. Data from two of the largest healthcare record databases in Japan—MDV and JMDC—were obtained for patients who visited a hospital for osteoporosis treatment from March 2018 to August 2021. In both databases, the demographic profiles of patients were similar for all cohorts. Most patients were elderly (mean age: 72.9–78.0 years) and female (> 80%), consistent with previous osteoporosis studies [27–29]. Most patients received their index treatment in an outpatient hospital setting. Overall, no significant differences in age and sex were observed in year-1 and year-2 romosozumab users. The prevalence of fracture history was higher in year-2 romosozumab users, while the prevalence of ischemic heart disease and cerebrovascular disease was lower among year-2 romosozumab users.

In this study, 24.4%–65.5% of patients with osteoporosis in the MDV and 27.8%–67.5% in the JMDC database reported osteoporotic fractures during the baseline period, of which vertebral fractures were the most common (58%–‍72%). This is consistent with the results of a multicenter, retrospective study conducted in Japan, which showed that 34.4% of patients with osteoporosis had at least one fracture in the past 5 years, of which 73% were vertebral [30]. This has been further corroborated by studies that have reported the vertebrae as the most common site of osteoporotic fractures [7, 8]. As a recommended first-line therapy for patients at risk of fracture, the current study used TPTD as a proxy for a population with a high risk of osteoporotic fracture [31]. Interestingly, a high proportion of patients in the romosozumab year-1 and year-2 cohorts and TPTD cohorts had fractures during the baseline period. The rate of vertebral fractures also remained consistent between these cohorts. In contrast, fewer patients in the non-TPTD cohort had a fracture history. These results suggest that both romosozumab and TPTD were prescribed for patients at high risk of fracture, indicating that the guidelines for osteoporosis treatment are largely followed in Japan. The prescription of romosozumab and TPTDs may reflect the high proportion of aged population in Japan, indicating that the risk of fracture is a primary consideration for patients with osteoporosis. Importantly, recent meta-analysis evidence has indicated that romosozumab demonstrates superior efficacy and fewer adverse effects among postmenopausal patients, particularly showing a significant improvement in lumbar spine and total hip BMD over 12 months, compared with TPTD (mean difference [95% CI]: 4.93, [4.21, 5.64] and 3.17 [2.68, 3.65], respectively) [32]. Similarly, trial results have shown greater improvements in spine and hip bone mineral content among postmenopausal patients treated with romosozumab compared with those treated with TPTD, suggesting that romosozumab may be preferable for rapidly reducing the risk of fracture [33]. Furthermore, computed tomography data of the vertebrae have shown that at 12 months, romosozumab increased cortical BMD by a mean of 2.1% from baseline, whereas TPTD led to a mean decrease of 0.1% [34]. These results suggest that romosozumab demonstrated greater spine and hip bone mineral density gain over TPTDs for patients with osteoporosis at high risk of fracture.

Little difference in baseline CV disease was found across cohorts, suggesting that physicians’ decisions to prescribe romosozumab, TPTD, or non-TPTD medications were not largely affected by patients’ CV history. In a double-blind randomized controlled trial (the ARCH study), an imbalance in positively adjudicated serious CV events was observed in patients receiving romosozumab (2.5%) and those receiving alendronate (1.9%) [35]. However, in a large placebo-controlled trial of romosozumab in postmenopausal women with osteoporosis (the FRAME study), CV events were similar between the romosozumab and placebo groups after 1 year of treatment [36], and the rates of CV events were not affected by romosozumab discontinuation. Additional data from a romosozumab safety report in Japan further demonstrated that the incidence of cerebrovascular disorders and ischemic heart disease in patients treated with romosozumab since March 4, 2019, and up to 1 year after launch was lower than that in the general population or in other related studies [19, 37, 38]. These findings suggest that the positively adjudicated CV event differences observed in the ARCH study were most likely not attributable to romosozumab [39]. Nonetheless, a warning has been issued in Japan for physicians regarding the prescription of romosozumab, stating that “the benefit of fracture reduction effect and CV risk with romosozumab should be sufficiently understood when patients are selected for romosozumab treatment. Prescribing romosozumab should be avoided at least in patients who have had ischemic heart disease or cerebrovascular disorder within the past year”. In the current study, although CV disease was generally balanced between cohorts following the addition of this warning to the romosozumab label in 2019, the incidence of angina pectoris and cerebral infarction was slightly lower in the year-2 cohort than in the year-1 cohort, suggesting that this addition may have impacted prescribing trends [20].

A recent analysis based on the Japanese Adverse Drug Event Report database (JADER) [40] reported a disproportionality of both cardiac and cerebrovascular events among romosozumab users only (n = 859). Results of multivariate logistic analysis showed significantly higher risk of cardiac events in romosozumab treated patients with cardiac disease (odds ratio [OR]: 5.9, 95% CI 3.5–9.9; P < 0.01) and hypertension (OR: 1.6, 95% CI 1.0–2.7; P = 0.047). Similarly, the risk of cerebrovascular events was significantly higher in patients treated with romosozumab in the presence of cerebrovascular disease (OR: 2.7, 95% CI 1.2–6.2; P = 0.02) and hypertension (OR: 2.6, 95% CI 1.7–3.9; P < 0.01). However, this paper mentioned the following limitations: Post approval of romosozumab by the Japanese Pharmaceutical and Medical Devices Agency in January 2019, the safety warnings were reported later in September 2019 for serious cardiac and cerebrovascular events. This warning may have enabled increased reporting of these adverse events making them more detectable. Further, the positive disproportionality could be attributed to the higher severity of osteoporosis and cardiovascular risk among patients treated with romosozumab. JADER allows for adjustment of limited covariates only (sex, age, cardiac disease, cerebrovascular disease, hypertension, and diabetes), as number of patients with other comorbidities (arrhythmia, chronic obstructive pulmonary disease, coronary artery disease, depression, obesity, peripheral vascular disease, and smoking) are limited. Additionally, for hypertension and diabetes, information on the severity of symptoms and duration of treatment is not available, suggesting that the present results may be influenced by the treatment status of these comorbidities. Also, disproportionality analysis can show an increase in frequency of certain adverse events only because JADER is a database of adverse events [40]. Morover, disproportionality analysis for this study applied the reporting odds ratio (ROR). ROR is calculated solely on the number of reported adverse events; it is essentially an indication of the intensity of the report of adverse events of interest reported for the target drug, and therefore it is not appropriate to compare the adverse events with other drugs. In addition, the risk management plan (RMP) has been applied to the drug on NDA since April, 2015, and ischemic diseases and cerebrovascular disorders are stated as potential risks associated with romosozumab, which was launched on March 4, 2019, in its RMP. The other recent report using TriNetX (a global federated health research network with real-time electronic medical records from 113 medical facilities from 16 countries at the time of analysis) enrolled patients aged ≥ 40 years, with a diagnosis of osteoporosis and a prescription of romosozumab or a PTH analog (teriparatide/abaloparatide) between August 2019 and August 2022. The study created a 1:1 propensity score–matched cohorts using demographic variables, comorbidities, and medications. Using Kaplan–Meier analysis, the incident 3-point major adverse CV event or death (3P-MACE) was significantly less frequent in the romosozumab (n = 158) vs. PTH analog (n = 211) cohort (p = 0.003), with reductions in myocardial ischemic events, cerebrovascular events, and deaths. The study concluded that in a diverse real-world setting, romosozumab prescribed for osteoporosis is associated with less adverse CV events compared with PTH analog therapy [41]. Limitations of the current study stem from its observational and retrospective design as well as from the inherent limitations of reliance on EMRs and propensity score matching. To test the robustness of our results, we used other known adverse effects of both medication categories as positive control outcomes. A unique potential bias in the case of the CV safety evaluation of romosozumab is the “healthy user bias” or the bias by indication. Though this was addressed in the study design, residual bias cannot be completely excluded; thus, we used “encounters for immunizations” as a negative control outcome. An additional limitation of the study was our inability to determine specific causes of death separating overall and cardiovascular specific mortality. As for generalizability, most of our cohorts were white non-Hispanic or Latino females from developed countries with a relatively low burden of CV risk factors. Thus, though our results are encouraging for the overall CV safety of romosozumab, they cannot be used to justify use of romosozumab in specific high CV risk situations and minority groups. To date, no prospective study was sufficiently powered to evaluate CV safety of romosozumab. Thus, prospective studies specifically designed and powered to evaluate the CV safety of romosozumab are still warranted, especially in older high CV risk patients. Finally, this is a retrospective database study for which comparisons are date/time/duration specific (initial landscape), and thus results should be cautiously interpreted for application in the current landscape.

Indeed, overall, patients who initiated romosozumab had lower rates of baseline CV-related risk factors such as diabetes and hyperlipidemia, possibly suggesting that physicians avoided prescribing romosozumab to patients with a history of CV risk factors. As this study includes a period both before and after this label change occurred, future work is needed to evaluate the impact of this change.

The most common comorbidities reported in this study were those expected in elderly patients, such as cardiometabolic diseases (hypertension, hyperlipidemia, diabetes, and chronic pulmonary disease), rheumatoid arthritis, and peptic ulcers. Angiotensin II receptor blockers, corticosteroids, cyclooxygenase-2-inhibitors, nonsteroidal antiinflammatory agents, anticoagulants, calcium channel blockers, and statins were the most common concomitant nonosteoporosis medications used by the patients in all cohorts, reflecting the management required for these comorbidities.

In a previous multicenter chart review study conducted in Japan, patients with osteoporosis were most commonly prescribed antiresorptive agents such as oral bisphosphonates (41.7%) and SERMs (16.1%), anabolic bone-forming agents such as TPTD (15.9%), active vitamin D_3_ (13.6%), and elcatonin/calcitonin (9.2%) [30]. In the current study, most patients who initiated romosozumab had received antiosteoporotic medication during the baseline period, of which active vitamin D_3_ (including alfacalcidol, calcitriol, and eldecalcitol), bisphosphonates, and TPTD were the most common. The baseline use of other antiosteoporotic medications was less common in the year-2 cohort. Among the patients who initiated TPTD, recombinant QD was the most frequent, followed by acetate QW. A higher proportion of patients in the TPTD group compared with those in the non-TPTD group had baseline fractures, likely reflecting the physician’s choice to treat such cases with TPTD. Previously, patients with a history of osteoporosis drug use have often been excluded from pivotal romosozumab studies [35, 42]. However, a real-world study conducted in Japan found that romosozumab treatment increased BMD from baseline regardless of previous treatment, with a slightly lower percentage change from baseline in patients who switched from another osteoporosis regimen compared with treatment-naive patients [43]. As percentage changes in BMD from baseline are affected by the baseline BMD value itself, future studies must assess the difference in baseline BMD when investigating the therapeutic effects of romosozumab in patients previously treated for osteoporosis.

The increasing administration of romosozumab may be influenced by certain factors promoting its accessibility. For example, the Central Social Insurance Medical Council (Chuikyo) approved romosozumab within their listings in February 2019. Additionally, the Amgen SupportPlus Co-Pay Program may support commercially insured patients by reducing the associated costs of romosozumab [44]. Moreover, a cost-effective analysis of romosozumab/alendronate versus TPTD/alendronate within the Japanese healthcare system has shown that romosozumab is substantially more cost-effective and results in a superior rate of quality-adjusted life-years [45]. In the current study, the mean number of romosozumab injections administered to patients over 12 months was similar in the year-1 (MDV: 9.7; JMDC: 7.3) and year-2 (MDV: 9.8; JMDC: 7.8) cohorts; as full compliance required 24 injections over 12 months, the treatment persistence rate at the 12 month follow-up was low for both cohorts (MDV: 37.6%, 42.9%; JMDC: 41.2%, 47.3%), whereas the mean adherence was similar (MDV: 71.0%, 71.0%; JMDC: 58.0%, 61.0%). In both cohorts, a higher rate of romosozumab injection administration was observed in the MDV database than in the JMDC database. This may be partially because the JMDC database sources health insurance data for company employees, leading to an underrepresentation of elderly individuals who are at a higher risk of fracture [46]. In contrast, compliance was lower in the MDV database, which, unlike the JMDC database, may reflect the inability to track patients across different institutions in this dataset [46]. Recently, Nakatoh et al. reported an antiosteoporotic pharmacotherapy persistence rate of 56.6% in the first year of treatment [16], whereas an adherence rate of 69.4% was reported in patients receiving monthly bisphosphonate treatment in a retrospective study in Japan [47]. TPTD (recombinant, once-daily self-injection) was also shown to have a year-1 mean MPR of 0.702 and persistence rate of 61% in Japan [48].

Real-world evidence is a valuable tool for studying patient characteristics outside the rigorous constraints of a clinical trial. The present study was observational in nature, providing real-world evidence from a more representative, unselected sample of patient population with osteoporosis. The large sample size of this study adds to the validity of the findings. However, such observational studies have inherent flaws, which include difficulties with data quality and consistency, as well as issues with missing data, underreporting, and coding errors. Furthermore, patients with osteoporosis at a high risk of fracture, particularly those with prevalent hip fractures and a high risk of subsequent fractures, are typically aged > 70 years and may not be fully covered by the databases assessed in this study. Lastly, owing to the nature of the data, patient data could not be traced if they switched hospitals. Nonetheless, the data presented in this study allow for the assessment of clinical characteristics and real-world treatment trends in Japanese patients with osteoporosis who are at a high risk of fracture. The outcomes data presented may guide future studies of romosozumab and TPTD, such as the CV history, which may inform the Pharmaceuticals and Medical Devices Agency medical information database network (MID-NET®).

This study provides real-world clinical characteristics and treatment patterns in patients with osteoporosis who received romosozumab or other antiosteoporosis drugs after romosozumab launch in Japan. Patients initiating romosozumab had higher rates of fracture history, similar to those initiating TPTD, than those initiating other antiosteoporotic medications; however, there was no apparent difference in CV history among treatment groups or databases. Romosozumab has been well accepted as a treatment option for patients with osteoporosis who are at a high risk of fracture, with appropriate use according to the indication since its launch in 2019 in Japan. First-line treatment of osteoporosis with an agent such as romosozumab, which exerts a dual effect of promoting bone formation and suppressing bone resorption, therefore, appears promising.

## Supplementary Information

Below is the link to the electronic supplementary material.

Supplementary file1 (DOCX 676 KB)

## Data Availability

The data are not publicly available.
