# Supplementary material for: Drug utilization pattern of romosozumab and other osteoporosis treatments in Japan, 2019–2021
Source: J Bone Miner Metab. 2024 Jul 10;42(6):653–67. doi: 10.1007/s00774-024-01530-6 (PMC11631996; doi:10.1007/s00774-024-01530-6)
Supplement: Supplementary file 1 — Supplementary file1 (DOCX 676 KB) [file 774_2024_1530_MOESM1_ESM.docx]

**Supplementary Information**

**Title:** A real-world, retrospective study of romosozumab and other antiosteoporotic treatments for Japanese patients with osteoporosis at high risk of fracture

**Journal name:** Journal of Bone and Mineral Metabolism

**Authors:** Satoshi Soen^a^, Alex Wang^b^, Etsuro Hamaya^c^, Hsu-Chih Chien^d^, Tzu-Chieh Lin^d^

**Affiliations:** ^a^Soen Orthopedics, Osteoporosis, and Rheumatology Clinic, Kobe, Japan; ^b^Medical Development, Amgen Inc., Sydney, Australia; ^c^Medical Affairs, Amgen K.K., Tokyo, Japan; ^d^Center for Observational Research, Amgen Inc., Thousand Oaks, California, USA

**Corresponding author:**

Etsuro Hamaya

Medical Affairs, Amgen K.K., Tokyo, Japan

Email: [ehamaya@amgen.com](mailto:ehamaya@amgen.com)

**Supplementary Information**

**Online Resource Fig. S1** Study design


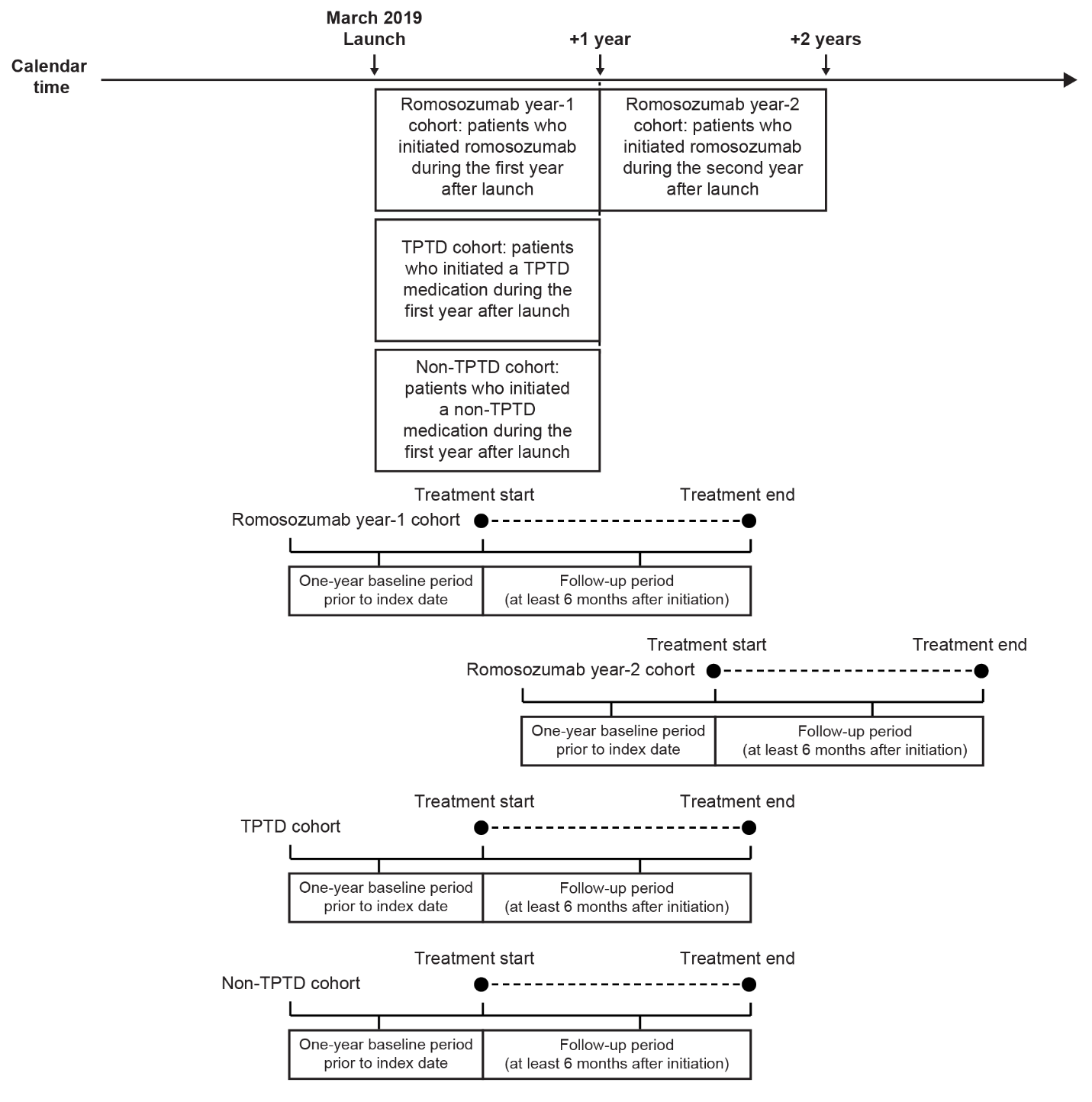


*TPTD* teriparatide

**Online Resource Fig. S2** Patient disposition


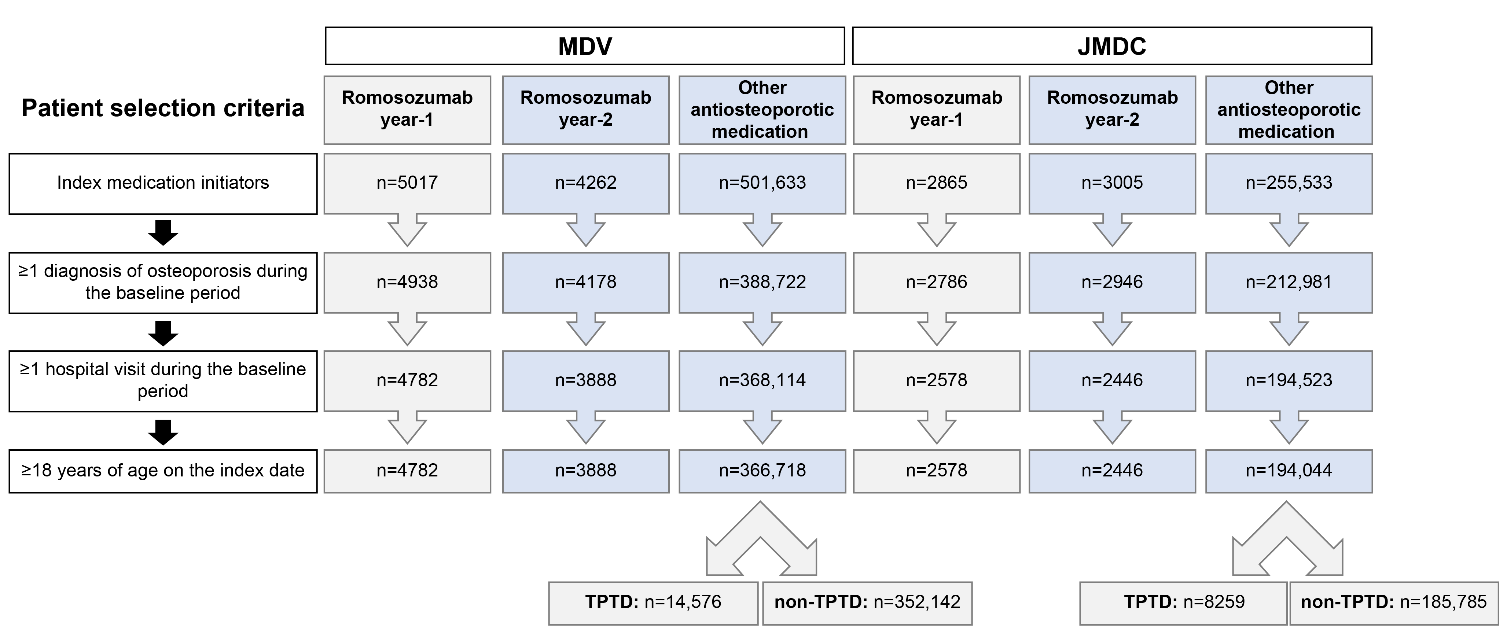


*JMDC* Japan Medical Data Center, *MDV* Medical Data Vision, *TPTD* teriparatide

**Online Resource Table S1** Baseline characteristics collected

| **Category** | **Description** |
| --- | --- |
| Demographic characteristics | Age, sex, type of hospital visit (inpatient or outpatient) |
| Osteoporotic fracture history | Previous fracture, site of fracture |
| Cardiovascular disease | Acute coronary syndrome (acute myocardial infarction, unstable angina), coronary revascularization (percutaneous coronary intervention, coronary artery bypass grafting), stroke (cerebral infarction, cerebral hemorrhage, subarachnoid hemorrhage), transient ischemic attack, and cerebral aneurysms |
| Comorbidities | HIV/AIDS, chronic obstructive pulmonary disease, chronic kidney disease, chronic kidney disease on dialysis, diabetes, diabetes complications, moderate or severe liver disease, asthma, cardiac arrhythmias, chronic pulmonary disease, congestive heart failure, dementia, hemiplegia or paraplegia, hyperlipidemia, hypertension, liver disease, Parkinson’s disease, peptic ulcer, peripheral vascular disease, rheumatoid arthritis, and systemic lupus erythematosus |
| Concomitant medications | Angiotensin II receptor blockers, angiotensin-converting enzyme inhibitors, anticoagulants, antidementia medications, antidepressants, antidiabetic medications, antiparkinsonian disease medications, antiplatelets, beta blockers, calcium channel blockers, cholesterol-lowering medications, cyclooxygenase-2 inhibitors, diuretics, hormone replacement therapy, hypnotics, insulin, nonsteroidal antiinflammatory drugs, opioids, lipid-lowering drugs, PCSK9 inhibitors, statins, statins plus ezetimibe combinations, and steroids |
| Other antiosteoporotic medications | Bisphosphonates, calcium, calcitonin, denosumab, TPTD (recombinant QD, acetate QW, acetate 2QW), ipriflavone, methenolone acetate, estrogens, SERM, active vitamin D_3_, and vitamin K |

*2QW* twice weekly, *AIDS* acquired immunodeficiency syndrome, *HIV* human immunodeficiency virus, *PCSK9* proprotein convertase subtilisin/kexin type 9, *QD* daily, *QW* weekly, *SERM* selective estrogen receptor modulator, *TPTD* teriparatide

**Online Resource Table S2** Breakdown of CV disease history during the baseline period and at the index date

|  | **Romosozumab year-1** | **Romosozumab year-2** | **Mean difference**  **(year-1 vs year-2) % (95% CI)** | **TPTD** | **Mean difference**  **(year-1 vs TPTD)**  **% (95% CI)** | **Non-TPTD** | **Mean difference (year-1 vs non-TPTD) % (95% CI)** |
| --- | --- | --- | --- | --- | --- | --- | --- |
| **MDV database** | | | | | | | |
| **Patients, N** | 4782 | 3888 |  | 14,576 |  | 352,142 |  |
| **Ischemic heart disease (total), n (%)** | 610 (12.8) | 347 (8.9) | 3.8 (2.5, 5.2) | 1871 (12.8) | 0.0 (−1.1, 1.0) | 49,990 (14.2) | −1.4 (−2.4, −0.5) |
| Angina pectoris | 549 (11.5) | 306 (7.9) | 3.3 (2.1, 4.5) | 1671 (11.5) | 0.0 (−1.1, 1.0) | 44,734 (12.7) | −1.1 (−2.0, −0.2) |
| Acute MI | 28 (0.6) | 15 (0.4) | 0.2 (−0.1, 0.5) | 95 (0.7) | −0.1 (−0.3, 0.2) | 2801 (0.8) | −0.2 (−0.4, 0.0) |
| Subsequent MI | NA | NA | NA | NA | NA | 8 (<0.1) | NA |
| Certain current complications following acute MI | 2 (<0.1) | NA | NA | 2 (<0.1) | 0.0 (0.0, 0.1) | 59 (<0.1) | 0.0 (0.0, 0.1) |
| Other acute ischemic heart diseases | 5 (0.1) | 3 (0.1) | 0.0 (−0.1, 0.2) | 24 (0.2) | −0.1 (−0.2, 0.1) | 473 (0.1) | 0.0 (−0.1, 0.1) |
| Chronic ischemic heart disease | 106 (2.2) | 61 (1.6) | 0.6 (0.0, 1.2) | 356 (2.4) | −0.2 (−0.7, 0.3) | 9632 (2.7) | −0.5 (−0.9, −0.1) |
| **Cerebrovascular disease (total), n (%)** | 673 (14.1) | 432 (11.1) | 3.0 (1.5, 4.4) | 1856 (12.7) | 1.3 (0.2, 2.5) | 49,019 (13.9) | 0.2 (−0.8, 1.2) |
| Subarachnoid hemorrhage | 13 (0.3) | 4 (0.1) | 0.2 (0.0, 0.4) | 37 (0.3) | 0.0 (−0.2, 0.2) | 879 (0.2) | 0.0 (−0.1, 0.2) |
| Intracerebral hemorrhage | 31 (0.6) | 22 (0.6) | 0.1 (−0.3, 0.4) | 80 (0.5) | 0.1 (−0.2, 0.4) | 2178 (0.6) | 0.0 (−0.1, 0.2) |
| Other nontraumatic intracranial hemorrhage | 19 (0.4) | 12 (0.3) | 0.1 (−0.2, 0.4) | 49 (0.3) | 0.1 (−0.2, 0.3) | 1020 (0.3) | 0.1 (−0.1, 0.3) |
| Cerebral infarction | 294 (6.1) | 182 (4.7) | 1.4 (0.4, 2.3) | 851 (5.8) | 0.3 (−0.5, 1.1) | 22,833 (6.5) | −0.3 (−1.0, 0.4) |
| Stroke, not specified as hemorrhage or infarction | 2 (0.0) | NA | NA | NA | NA | 40 (<0.1) | 0.0 (−0.0, 0.1) |
| Occlusion and stenosis of precerebral arteries, not resulting in cerebral infarction | 8 (0.2) | 2 (0.1) | 0.1 (−0.0, 0.3) | 14 (0.1) | 0.1 (−0.1, 0.2) | 228 (0.1) | 0.1 (0.0, 0.2) |
| Occlusion and stenosis of cerebral arteries, not resulting in cerebral infarction | 115 (2.4) | 42 (1.1) | 1.3 (0.7, 1.8) | 307 (2.1) | 0.3 (−0.2, 0.8) | 9378 (2.7) | −0.3 (−0.7, 0.2) |
| Other cerebrovascular diseases | 107 (2.2) | 72 (1.9) | 0.4 (−0.2, 1.0) | 251 (1.7) | 0.5 (0.0, 1.0) | 6891 (2.0) | 0.3 (−0.2, 0.7) |
| Sequelae of cerebrovascular disease | 250 (5.2) | 169 (4.3) | 0.8 (−0.1, 1.7) | 760 (5.2) | 0.0 (−0.7, 0.7) | 17,134 (4.9) | 0.3 (−0.3, 1.0) |
| Transient cerebral ischemic attacks and related syndromes | 43 (0.9) | 15 (0.4) | 0.5 (0.2, 0.9) | 72 (0.5) | 0.4 (0.1, 0.7) | 2462 (0.7) | 0.2 (−0.1, 0.5) |
| **JMDC database** | | | | | | | |
| **Patients, N** | 2578 | 2446 |  | 8259 |  | 185,785 |  |
| **Ischemic heart disease (total), n (%)** | 327 (12.7) | 277 (11.3) | 1.4 (−0.5, 3.2) | 1082 (13.1) | −0.4 (−1.9, 1.1) | 28,770 (15.5) | −2.8 (−4.1, −1.5) |
| Angina pectoris | 303 (11.8) | 244 (10.0) | 1.6 (−0.1, 3.3) | 957 (11.6) | 0.1 (−1.2, 1.5) | 25,753 (13.9) | −1.9 (−3.1, −0.7) |
| Acute MI | 9 (0.3) | 5 (0.2) | 0.1 (−0.2, 0.5) | 49 (0.6) | −0.2 (−0.5, 0.1) | 1346 (0.7) | −0.4 (−0.6, −0.1) |
| Subsequent MI | NA | NA | NA | NA | NA | 3 (<0.1) | NA |
| Certain current complications following acute MI | 1 (<0.1) | NA | NA | 1 (<0.1) | 0.0 (−0.1, 0.1) | 32 (<0.1) | 0.0 (−0.1, 0.1) |
| Other acute ischemic heart diseases | 3 (0.1) | 2 (0.1) | 0.0 (−0.2, 0.2) | 8 (0.1) | 0.0 (−0.2, 0.2) | 211 (0.1) | 0.0 (−0.1, 0.2) |
| Chronic ischemic heart disease | 45 (1.7) | 51 (2.1) | −0.3 (−1.1, 0.5) | 202 (2.4) | −0.7 (−1.3, −0.1) | 5346 (2.9) | −1.1 (−1.6, −0.6) |
| **Cerebrovascular diseases (total), n (%)** | 345 (13.4) | 308 (12.6) | 0.8 (−1.1, 2.7) | 1148 (13.9) | −0.5 (−2.1, 1.0) | 29,018 (15.6) | −2.2 (−3.6, −0.9) |
| Subarachnoid hemorrhage | 4 (0.2) | 6 (0.2) | −0.1 (−0.4, 0.2) | 17 (0.2) | −0.1 (−0.3, 0.2) | 463 (0.2) | −0.1(−0.3, 0.1) |
| Intracerebral hemorrhage | 11 (0.4) | 15 (0.6) | −0.2 (−0.6, 0.3) | 43 (0.5) | −0.1 (−0.4, 0.2) | 1236 (0.7) | −0.2 (−0.5, 0.0) |
| Other nontraumatic intracranial hemorrhage | 4 (0.2) | 4 (0.2) | 0.0 (−0.3, 0.3) | 31 (0.4) | −0.2 (−0.4, 0.0) | 609 (0.3) | −0.2 (−0.3, 0.0) |
| Cerebral infarction | 156 (6.1) | 113 (4.6) | 1.3 (0.1, 2.6) | 455 (5.5) | 0.5 (−0.5, 1.6) | 13,221 (7.1) | −1.0 (−1.9, −0.1) |
| Stroke, not specified as hemorrhage or infarction | NA | NA | NA | 2 (<0.1) | NA | 22 (<0.1) | NA |
| Occlusion and stenosis of precerebral arteries, not resulting in cerebral infarction | 53 (2.1) | 38 (1.6) | 0.5 (−0.3, 1.3) | 139 (1.7) | 0.4 (−0.3, 1.0) | 4880 (2.6) | −0.6 (−1.1, 0.0) |
| Occlusion and stenosis of cerebral arteries, not resulting in cerebral infarction | 14 (0.5) | 10 (0.4) | 0.1 (−0.3, 0.6) | 47 (0.6) | 0.0 (−0.4, 0.3) | 1271 (0.7) | −0.1 (−0.4, 0.2) |
| Other cerebrovascular diseases | 51 (2.0) | 46 (1.9) | 0.1 (−0.7, 0.9) | 156 (1.9) | 0.1 (−0.5, 0.7) | 3793 (2.0) | −0.1 (−0.6, 0.5) |
| Sequelae of cerebrovascular disease | 134 (5.2) | 120 (4.9) | 0.3 (−1.0, 1.5) | 525 (6.4) | −1.1 (−2.1, −0.1) | 10,354 (5.6) | −0.4 (−1.2, 0.5) |
| Transient cerebral ischemic attacks and related syndromes | 7 (0.3) | 23 (0.9) | −0.7 (−1.1, −0.2) | 52 (0.6) | −0.4 (−0.6, −0.1) | 1337 (0.7) | −0.4 (−0.7, −0.2) |

*CI* confidence interval, *CV* cardiovascular, *JMDC* Japan Medical Data Center, *MDV* Medical Data Vision, *MI* myocardial infarction, *NA* not available, *PCI* percutaneous coronary intervention, *TPTD* teriparatide

**Online Resource Table S3** Other concomitant medication use during the baseline period

|  | **Romosozumab year-1** | **Romosozumab year-2** | **Mean difference (year-1 vs year-2)**  **% (95% CI)** | **TPTD** | **Mean difference**  **(year-1 vs TPTD)**  **% (95% CI)** | **Non-TPTD** | **Mean difference**  **(year-1 vs non-TPTD) % (95% CI)** |
| --- | --- | --- | --- | --- | --- | --- | --- |
| **MDV database** | | | | | | | |
| **Anticoagulants** | 481 (10.1) | 338 (8.7) | 1.4 (0.1, 2.6) | 1964 (13.5) | −3.4 (−4.4, −2.4) | 48,469 (13.8) | −3.7 (−4.6, −2.8) |
| **Antidementia medications** | 105 (2.2) | 69 (1.8) | 0.4 (−0.2, 1.0) | 338 (2.3) | −0.1 (−0.6, 0.4) | 8633 (2.5) | −0.3 (−0.7, 0.2) |
| **Antidepressants** | 235 (4.9) | 191 (4.9) | 0.0 (−0.9, 0.9) | 833 (5.7) | −0.8 (−1.5, −0.1) | 12,906 (3.7) | 1.2 (0.6, 1.9) |
| **Antidiabetic agents (any of below)** | 271 (5.7) | 184 (4.7) | 0.9 (−0.0, 1.9) | 1048 (7.2) | −1.5 (−2.3, −0.7) | 33,584 (9.5) | −3.9 (−4.5, −3.2) |
| Insulin | 94 (2.0) | 77 (2.0) | −0.0 (−0.6, 0.6) | 497 (3.4) | −1.4 (−1.9, −0.9) | 13,216 (3.8) | −1.8 (−2.2, −1.4) |
| Blood glucose–lowering agents | 223 (4.7) | 146 (3.8) | 0.9 (0.0, 1.8) | 835 (5.7) | −1.1 (−1.8, −0.3) | 27,270 (7.7) | −3.1 (−3.7, −2.5) |
| **Antihypertensive agents (any of below)** | 1028 (21.5) | 784 (20.2) | 1.3 (−0.4, 3.1) | 3673 (25.2) | −3.7 (−5.1, −2.3) | 114,701 (32.6) | −11.1 (−12.3, −9.9) |
| Angiotensin II receptor blockers | 467 (9.8) | 337 (8.7) | 1.1 (−0.1, 2.3) | 1710 (11.7) | −2.0 (−3.0, −1.0) | 58,097 (16.5) | −6.7 (−7.6, −5.9) |
| Angiotensin-converting enzyme inhibitors | 39 (0.8) | 22 (0.6) | 0.2 (−0.1, 0.6) | 131 (0.9) | −0.1 (−0.4, 0.2) | 4815 (1.4) | −0.6 (−0.8, −0.3) |
| Beta blockers | 233 (4.9) | 154 (4.0) | 0.9 (0.0, 1.8) | 833 (5.7) | −0.8 (−1.6, −0.1) | 24,939 (7.1) | −2.2 (−2.8, −1.6) |
| Calcium channel blockers | 598 (12.5) | 472 (12.1) | 0.4 (−1.0, 1.8) | 2176 (14.9) | −2.4 (−3.5, −1.3) | 63,670 (18.1) | −5.6 (−6.5, −4.6) |
| Thiazide diuretics | 34 (0.7) | 15 (0.4) | 0.3 (−0.0, 0.7) | 184 (1.3) | −0.6 (−0.9, −0.2) | 5093 (1.4) | −0.7 (−1.0, −0.5) |
| Other diuretics | 264 (5.5) | 181 (4.7) | 0.9 (−0.1, 1.8) | 901 (6.2) | −0.7 (−1.4, 0.1) | 28,935 (8.2) | −2.7 (−3.4, −2.0) |
| **Antiparkinsonian agents** | 107 (2.2) | 75 (1.9) | 0.3 (−0.3, 0.9) | 448 (3.1) | −0.8 (−1.4, −0.3) | 8825 (2.5) | −0.3 (−0.7, 0.2) |
| **Antiplatelets** | 313 (6.5) | 182 (4.7) | 1.9 (0.9, 2.9) | 1070 (7.3) | −0.8 (−1.6, 0.0) | 35,429 (10.1) | −3.5 (−4.2, −2.8) |
| **Corticosteroid** | 658 (13.8) | 512 (13.2) | 0.6 (−0.9, 2.1) | 1904 (13.1) | 0.7 (−0.4, 1.8) | 113,260 (32.2) | −18.4 (−19.4, −17.4) |
| **Cyclooxygenase-2 inhibitors** | 562 (11.8) | 564 (14.5) | −2.8 (−4.2, −1.3) | 2079 (14.3) | −2.5 (−3.6, −1.4) | 26,210 (7.4) | 4.3 (3.4, 5.2) |
| **Hormone replacement therapy** | 2 (0.0) | 4 (0.1) | −0.1 (−0.2, 0.1) | 3 (0.0) | 0.0 (−0.1, 0.1) | 945 (0.3) | −0.2 (−0.3, −0.2) |
| **Hypnotics** | 457 (9.6) | 341 (8.8) | 0.8 (−0.5, 2.0) | 1777 (12.2) | −2.6 (−3.6, −1.6) | 44,898 (12.7) | −3.2 (−4.0, −2.3) |
| **Lipid-lowering agents (any of below)** | 476 (10.0) | 339 (8.7) | 1.2 (−0.0, 2.5) | 1698 (11.6) | −1.7 (−2.7, −0.7) | 66,71 (18.9) | −9.0 (−9.9, −8.1) |
| Statins | 450 (9.4) | 315 (8.1) | 1.3 (0.1, 2.5) | 1600 (11.0) | −1.6 (−2.6, −0.6) | 62,656 (17.8) | −8.4 (−9.2, −7.5) |
| Ezetimibe | 33 (0.7) | 31 (0.8) | −0.1 (−0.5, 0.3) | 114 (0.8) | −0.1 (−0.4, 0.2) | 5353 (1.5) | −0.8 (−1.1, −0.6) |
| PCSK9 | NA | NA | NA | 2 (0.0) | NA | 78 (0.0) | NA |
| Other lipid-lowering drugs | 5 (0.1) | 6 (0.2) | −0.0 (−0.2, 0.1) | 31 (0.2) | −0.1 (−0.2, 0.0) | 1047 (0.3) | −0.2 (−0.3, −0.1) |
| **Nonsteroidal antiinflammatory agents** | 937 (19.6) | 718 (18.5) | 1.1 (−0.6, 2.8) | 3172 (21.8) | −2.2 (−3.5, −0.8) | 63,438 (18.0) | 1.6 (0.4, 2.7) |
| **Opioids** | 80 (1.7) | 94 (1.7) | −0.7 (−1.4, −0.1) | 345 (2.4) | −0.7 (−1.1, −0.2) | 6052 (1.7) | −0.0 (−0.4, 0.3) |
| **JMDC database** | | | | | | | |
| **Anticoagulants** | 300 (11.6) | 245 (10.0) | 1.6 (−0.1, 3.4) | 1373 (16.6) | −5.0 (−6.5, −3.5) | 26,233 (14.1) | −2.5 (−3.8, −1.2) |
| **Antidementia medications** | 83 (3.2) | 61 (2.5) | 0.7 (−0.2, 1.7) | 267 (3.2) | −0.0 (−0.8, 0.8) | 5756 (3.1) | 0.1 (−0.6, 0.8) |
| **Antidepressants** | 147 (5.7) | 110 (4.5) | 1.2 (−0.0, 2.5) | 507 (6.1) | −0.4 (−1.5, 0.6) | 6899 (3.7) | 2.0 (1.1, 2.9) |
| **Antidiabetic agents (any of below)** | 140 (5.4) | 144 (5.9) | −0.5 (−1.8, 0.9) | 645 (7.8) | −2.4 (−3.5, −1.3) | 17,623 (9.5) | −4.1 (−5.0, −3.2) |
| Insulin | 58 (2.2) | 43 (1.8) | 0.5 (−0.3, 1.3) | 323 (3.9) | −1.7 (−2.4, −0.9) | 6780 (3.6) | −1.4 (−2.0, −0.8) |
| Blood glucose–lowering agents | 106 (4.1) | 122 (5.0) | −0.9 (−2.1, 0.3) | 493 (6.0) | −1.9 (−2.8, −0.9) | 14,224 (7.7) | −3.5 (−4.3, −2.7) |
| **Antihypertensive agents (any of below)** | 628 (24.4) | 562 (23.0) | 1.4 (−1.0, 3.8) | 2408 (29.2) | −4.8 (−6.7, −2.8) | 64,638 (34.8) | −10.4 (−12.1, −8.7) |
| Angiotensin II receptor blockers | 281 (10.9) | 265 (10.8) | 0.1 (−1.7, 1.8) | 1068 (12.9) | −2.0 (−3.5, −0.6) | 32,792 (17.7) | −6.8 (−8.0, −5.5) |
| Angiotensin-converting enzyme inhibitors | 21 (0.8) | 30 (1.2) | −0.4 (−1.0, 0.2) | 97 (1.2) | −0.4 (−0.8, 0.1) | 2659 (1.4) | −0.6 (−1.0, −0.2) |
| Beta blockers | 109 (4.2) | 105 (4.3) | −0.1 (−1.2, 1.1) | 487 (5.9) | −1.7 (−2.6, −0.7) | 13,651 (7.3) | −3.1 (−3.9, −2.3) |
| Calcium channel blockers | 386 (15.0) | 322 (13.2) | 1.8 (−0.2, 3.8) | 1479 (17.9) | −2.9 (−4.6, −1.3) | 36,423 (19.6) | −4.6 (−6.0, −3.2) |
| Thiazide diuretics | 30 (1.2) | 23 (0.9) | 0.2 (−0.4, 0.8) | 109 (1.3) | −0.2 (−0.7, 0.4) | 2901 (1.6) | −0.4 (−0.8, 0.0) |
| Other diuretics | 152 (5.9) | 143 (5.8) | 0.0 (−1.3, 1.4) | 632 (7.7) | −1.8 (−2.9, −0.7) | 16,717 (9.0) | −3.1 (−4.0, −2.2) |
| Antiparkinsonian agents | 72 (2.8) | 48 (2.0) | 0.8 (−0.1, 1.7) | 297 (3.6) | −0.8 (−1.6, −0.0) | 5072 (2.7) | 0.1 (−0.6, 0.7) |
| Antiplatelets | 157 (6.1) | 140 (5.7) | 0.4 (−1.0, 1.7) | 635 (7.7) | −1.6 (−2.7, −0.5) | 20,656 (11.1) | −5.0 (−6.0, −4.1) |
| Corticosteroid | 293 (11.4) | 281 (11.5) | −0.1 (−1.9, 1.7) | 1071 (13.0) | −1.6 (−3.1, −0.2) | 52,106 (28.0) | −16.7 (−17.9, −15.4) |
| Cyclooxygenase-2 inhibitors | 382 (14.8) | 360 (14.7) | 0.1 (−1.9, 2.1) | 1401 (17.0) | −2.1 (−3.8, −0.5) | 14,796 (8.0) | 6.9 (5.5, 8.3) |
| Hormone replacement therapy | NA | 1 (0.0) | NA | 2 (0.0) | NA | 415 (0.2) | NA |
| Hypnotics | 322 (12.5) | 256 (10.5) | 2.0 (0.2, 3.8) | 1344 (16.3) | −3.8 (−5.3, −2.3) | 24,885 (13.4) | −0.9 (−2.2, 0.4) |
| **Lipid-lowering agents (any of below)** | 283 (11.0) | 249 (10.2) | 0.8 (−0.9, 2.5) | 962 (11.6) | −0.7 (−2.1, 0.7) | 36,106 (19.4) | −8.5 (−9.7, −7.2) |
| Statins | 259 (10.0) | 229 (9.4) | 0.7 (−1.0, 2.4) | 913 (11.1) | −1.0 (−2.4, 0.4) | 33,941 (18.3) | −8.2 (−9.4, −7.0) |
| Ezetimibe | 23 (0.9) | 22 (0.9) | −0.0 (−0.6, 0.6) | 52 (0.6) | 0.3 (−0.2, 0.7) | 2787 (1.5) | −0.6 (−1.0, −0.2) |
| PCSK9 | NA | NA | NA | NA | NA | 35 (0.0) | NA |
| Other lipid-lowering drugs | 6 (0.2) | 5 (0.2) | 0.0 (−0.3, 0.3) | 19 (0.2) | 0.0 (−0.2, 0.2) | 608 (0.3) | −0.1 (−0.3, 0.1) |
| **Nonsteroidal antiinflammatory agents** | 575 (22.3) | 504 (20.6) | 1.7 (−0.6, 4.0) | 2049 (24.8) | −2.5 (−4.4, −0.6) | 36,410 (19.6) | 2.7 (1.1, 4.3) |
| **Opioids** | 135 (5.2) | 103 (4.2) | 1.0 (−0.2, 2.2) | 702 (8.5) | −3.3 (−4.3, −2.2) | 5709 (3.1) | 2.2 (1.3, 3.0) |

*CI* confidence interval, *JMDC* Japan Medical Data Center, *MDV* Medical Data Vision, *NA* not available, *PCSK9* proprotein convertase subtilisin/kexin type 9, *TPTD* teriparatide

**Online Resource Table S4** Duration and supply of antiosteoporotic medication use in the romosozumab cohort during the baseline period

| **Medications^a^** | **Romosozumab year-1** | | **Romosozumab year-2** | | **Mean difference** (**year-1 vs year-2)**  **% (95% CI)** | |
| --- | --- | --- | --- | --- | --- | --- |
|  | **MDV** | **JMDC** | **MDV** | **JMDC** | **MDV** | **JMDC** |
| **Patients, N** | 4782 | 2578 | 3888 | 2446 |  |  |
| **Osteoporotic medication use, n (%)** | 3064 (64.1) | 1430 (55.5) | 2357 (60.6) | 1220 (49.9) |  |  |
| **Days from initiation to index date, mean (SD)** | | | | | | |
| Bisphosphonate | 173.8 (102.8) | 168.8 (103.4) | 174.0 (104.5) | 172.5 (104.3) | −0.2 (−3.9, 3.5) | −3.7 (−10.4, 3.1) |
| Calcitonin | 141.1 (103.4) | 104.9 (89.0) | 147.3 (110.0) | 119.9 (107.2) | −6.2 (−22.0, 9.6) | −15.0 (−31.0, 1.0) |
| Calcium | 143.3 (99.8) | 122.9 (95.6) | 171.4 (121.9) | 112.5 (104.5) | −28.1 (−39.0, −17.3) | 10.3 (−2.2, 22.8) |
| Denosumab | 212.5 (89.5) | 191.3 (91.3) | 221.5 (87.6) | 200.8 (95.0) | −9.0 (−20.3, 2.3) | −9.5 (−30.1, 11.0) |
| Methenolone acetate | NA | 55.4 (63.9) | 116.5 (54.3) | NA | NA | NA |
| Estrogen | 160.9 (83.7) | 27.0 (NA) | 123.8 (98.5) | 187.3 (99.8) | 37.2 (−29.9, 104.2) | −160.3 (−437.5, 116.8) |
| Ipriflavone | 170.6 (112.0) | - | NA |  | NA |  |
| SERM | 167.2 (106.4) | 145.6 (103.9) | 177.3 (107.4) | 168.7 (111.5) | −10.1 (−18.1, −2.2) | −23.1 (−34.6, −11.5) |
| TPTD (any of the below) | 162.4 (106.6) | 156.5 (103.5) | 158.9 (104.7) | 157.7 (106.3) | 3.5 (0.0, 7.1) | −1.1 (−5.5, 3.2) |
| Recombinant QD | 164.1 (107.4) | 145.2 (104.4) | 149.9 (102.9) | 145.4 (106.2) | 14.3 (9.2, 19.3) | −0.2 (−6.5, 6.1) |
| Acetate QW | 160.8 (105.8) | 163.8 (102.3) | 173.6 (105.1) | 180.6 (103.7) | −12.8 (−17.8, −7.8) | −16.9 (−23.2, −10.5) |
| Acetate 2QW | 27.0 (NA) | 14.0 (12.5) | 78.7 (66.5) | 115.7 (86.9) | −51.7 (−183.1, 79.7) | −101.7 (−201.4, −2.0) |
| Active vitamin D_3_^c^ | 140.9 (103.7) | 126.5 (100.8) | 137.2 (106.0) | 126.7 (104.5) | 3.7 (1.1, 6.4) | −0.2 (−3.7, 3.3) |
| Vitamin K | 154.0 (115.7) | 151.8 (112.0) | 143.2 (112.5) | 132.4 (102.0) | 10.8 (0.0, 21.7) | 19.4 (2.4, 36.4) |
| **Days of supply, mean (SD)** | | | | | | |
| Bisphosphonate | 59.8 (90.4) | 49.1 (86.5) | 57.6 (96.9) | 44.1 (81.1) | 2.2 (−5.3, 9.8) | 4.9 (−6.4, 16.2) |
| Calcitonin | 35.1 (44.1) | 25.8 (30.1) | 23.9 (28.2) | 23.6 (28.9) | 11.2 (−0.5, 22.8) | 2.2 (−7.2, 11.6) |
| Calcium | 131.0 (130.1) | 117.8 (113.3) | 160.7 (137.2) | 134.9 (139.5) | −29.7 (−60.1, 0.7) | −17.2 (−64.0, 29.7) |
| Denosumab | 227.5 (82.4) | 218.3 (72.2) | 235.3 (85.1) | 223.9 (76.6) | −7.8 (−20.5, 4.9) | −5.6 (−25.4, 14.2) |
| Methenolone acetate | NA | 137.0 (142.8) | 84.3 (119.7) | NA | NA | NA |
| Estrogen | 100.3 (94.7) | 2.0 (NA) | 61.8 (112.4) | 12.0 (2.8) | 38.4 (−119.5, 196.3) | −10.0 (−54.0, 34.0) |
| Ipriflavone | 236.0 (131.2) | - | NA |  | NA |  |
| SERM | 208.0 (121.8) | 183.6 (120.2) | 221.7 (120.1) | 189.6 (119.2) | −13.7 (−36.5, 9.1) | −6.1 (−39.6, 27.4) |
| TPTD (any of the below) | 126.4 (102.7) | 137.6 (109.1) | 117.5 (106.1) | 125.9 (107.8) | 8.9 (−3.5, 21.3) | 11.7 (−6.0, 29.3) |
| Recombinant QD | 131.8 (93.2) | 133.3 (91.7) | 133.9 (94.6) | 143.9 (96.9) | −2.1 (−16.8, 12.7) | −10.6 (−31.0, 9.9) |
| Acetate QW | 118.3 (115.8) | 145.0 (125.3) | 130.1 (121.0) | 135.6 (123.7) | −11.8 (−35.7, 12.1) | 9.4 (−25.2, 44.3) |
| Acetate 2QW | 3.5 (NA) | 3.0 (0.9) | 12.4 (12.6) | 13.4 (12.9) | −8.9 (−34.3, 16.5) | −10.4 (−25.7, 5.0) |
| Active vitamin D_3_^c^ | 166.1 (130.2) | 130.6 (125.3) | 159.3 (134.3) | 133.3 (127.4) | 6.8 (−2.0, 15.5) | −2.7 (−14.4, 9.1) |
| Vitamin K | 205.5 (135.3) | 182.1 (134.4) | 165.1 (137.6) | 143.9 (131.4) | 40.3 (4.8, 75.9) | 38.2 (−26.7, 103.0) |

*2QW* twice weekly, *CI* confidence interval, *JMDC* Japan Medical Data Center, *MDV* Medical Data Vision, *NA* not available, *QD* daily, *QW* weekly, *SD* standard deviation, *SERM* selective estrogen receptor modulator, *TPTD* teriparatide

^a^The medication may be used as monotherapy or in combination with other antiosteoporotic medications

^b^Percentages calculated using the total number of patients who received osteoporotic medication as the denominator

^c^Vitamin D3 included alfacalcidol, calcitriol, and eldecalcitol
